# Effects of eight neuropsychiatric copy number variants on human brain structure

**DOI:** 10.1038/s41398-021-01490-9

**Published:** 2021-07-20

**Authors:** Claudia Modenato, Kuldeep Kumar, Clara Moreau, Sandra Martin-Brevet, Guillaume Huguet, Catherine Schramm, Martineau Jean-Louis, Charles-Olivier Martin, Nadine Younis, Petra Tamer, Elise Douard, Fanny Thébault-Dagher, Valérie Côté, Audrey-Rose Charlebois, Florence Deguire, Anne M. Maillard, Borja Rodriguez-Herreros, Aurèlie Pain, Sonia Richetin, 16p11.2 European Consortium, Marie-Claude Addor, Joris Andrieux, Benoît Arveiler, Geneviève Baujat, Frédérique Sloan-Béna, Marco Belfiore, Dominique Bonneau, Sonia Bouquillon, Odile Boute, Alfredo Brusco, Tiffany Busa, Jean- Hubert Caberg, Dominique Campion, Vanessa Colombert, Marie-Pierre Cordier, Albert David, François-Guillaume Debray, Marie-Ange Delrue, Martine Doco-Fenzy, Ulrike Dunkhase-Heinl, Patrick Edery, Christina Fagerberg, Laurence Faivre, Francesca Forzano, David Genevieve, Marion Gérard, Daniela Giachino, Agnès Guichet, Olivier Guillin, Delphine Héron, Bertrand Isidor, Aurélia Jacquette, Sylvie Jaillard, Hubert Journel, Boris Keren, Didier Lacombe, Sébastien Lebon, Cédric Le Caignec, Marie-Pierre Lemaître, James Lespinasse, Michèle Mathieu-Dramart, Sandra Mercier, Cyril Mignot, Chantal Missirian, Florence Petit, Kristina Pilekær Sørensen, Lucile Pinson, Ghislaine Plessis, Fabienne Prieur, Alexandre Raymond, Caroline Rooryck-Thambo, Massimiliano Rossi, Damien Sanlaville, Britta Schlott Kristiansen, Caroline Schluth-Bolard, Marianne Till, Mieke Van Haelst, Lionel Van Maldergem, Simons Searchlight Consortium, Hanalore Alupay, Benjamin Aaronson, Sean Ackerman, Katy Ankenman, Ayesha Anwar, Constance Atwell, Alexandra Bowe, Arthur L. Beaudet, Marta Benedetti, Jessica Berg, Jeffrey Berman, Leandra N. Berry, Audrey L. Bibb, Lisa Blaskey, Jonathan Brennan, Christie M. Brewton, Randy Buckner, Polina Bukshpun, Jordan Burko, Phil Cali, Bettina Cerban, Yishin Chang, Maxwell Cheong, Vivian Chow, Zili Chu, Darina Chudnovskaya, Lauren Cornew, Corby Dale, John Dell, Allison G. Dempsey, Trent Deschamps, Rachel Earl, James Edgar, Jenna Elgin, Jennifer Endre Olson, Yolanda L. Evans, Anne Findlay, Gerald D. Fischbach, Charlie Fisk, Brieana Fregeau, Bill Gaetz, Leah Gaetz, Silvia Garza, Jennifer Gerdts, Orit Glenn, Sarah E. Gobuty, Rachel Golembski, Marion Greenup, Kory Heiken, Katherine Hines, Leighton Hinkley, Frank I. Jackson, Julian Jenkins, Rita J. Jeremy, Kelly Johnson, Stephen M. Kanne, Sudha Kessler, Sarah Y. Khan, Matthew Ku, Emily Kuschner, Anna L. Laakman, Peter Lam, Morgan W. Lasala, Hana Lee, Kevin LaGuerre, Susan Levy, Alyss Lian Cavanagh, Ashlie V. Llorens, Katherine Loftus Campe, Tracy L. Luks, Elysa J. Marco, Stephen Martin, Alastair J. Martin, Gabriela Marzano, Christina Masson, Kathleen E. McGovern, Rebecca McNally Keehn, David T. Miller, Fiona K. Miller, Timothy J. Moss, Rebecca Murray, Srikantan S. Nagarajan, Kerri P. Nowell, Julia Owen, Andrea M. Paal, Alan Packer, Patricia Z. Page, Brianna M. Paul, Alana Peters, Danica Peterson, Annapurna Poduri, Nicholas J. Pojman, Ken Porche, Monica B. Proud, Saba Qasmieh, Melissa B. Ramocki, Beau Reilly, Timothy P. L. Roberts, Dennis Shaw, Tuhin Sinha, Bethanny Smith-Packard, Anne Snow Gallagher, Vivek Swarnakar, Tony Thieu, Christina Triantafallou, Roger Vaughan, Mari Wakahiro, Arianne Wallace, Tracey Ward, Julia Wenegrat, Anne Wolken, Lester Melie-Garcia, Leila Kushan, Ana I. Silva, Marianne B. M. van den Bree, David E. J. Linden, Michael J. Owen, Jeremy Hall, Sarah Lippé, Mallar Chakravarty, Danilo Bzdok, Carrie E. Bearden, Bogdan Draganski, Sébastien Jacquemont

**Affiliations:** 1 LREN - Department of Clinical Neurosciences, Centre Hospitalier Universitaire Vaudois and University of Lausanne, Lausanne, Switzerland; 2 Centre de recherche CHU Sainte-Justine and University of Montréal, Montréal, Canada; 3 Service des Troubles du Spectre de l’Autisme et apparentés, Centre Hospitalier Universitaire Vaudois and University of Lausanne, Lausanne, Switzerland; 4 Applied Signal Processing Group (ASPG), Swiss Federal Institute Lausanne (EPFL), Lausanne, Switzerland; 5 Semel Institute for Neuroscience and Human Behavior, Departments of Psychiatry and Biobehavioral Sciences and Psychology, UCLA, Los Angeles, USA; 6 School for Mental Health and Neuroscience, Maastricht University, Maastricht, Netherlands; 7 MRC Centre for Neuropsychiatric Genetics and Genomics, Cardiff University, Cardiff, UK; 8 Division of Psychological Medicine and Clinical Neurosciences, School of Medicine, Cardiff University, Cardiff, UK; 9 Neuroscience and Mental Health Research Institute, Cardiff University, Cardiff, UK; 10 Douglas Research Centre, McGill University, Montréal, QC Canada; 11 Department of Biomedical Engineering, McConnell Brain Imaging Centre; Montreal Neurological Institute, McGill University, Montréal, QC Canada; 12 Mila - Quebec Artificial Intelligence Institute, Montréal, QC Canada; 13 Neurology Department, Max-Planck-Institute for Human Cognitive and Brain Sciences, Leipzig, Germany; 14 Service de génétique médicale, Centre Hospitalier Universitaire Vaudois, Lausanne University, Lausanne, Switzerland; 15 Institut de Génétique Médicale, CHRU de Lille, Hopital Jeanne de Flandre, Lille, France; 16 Service de génétique médicale, CHU de Bordeaux- GH Pellegrin, Bordeaux, France; 17 Service de Génétique Médicale, CHU Paris - Hôpital Necker-Enfants Malades, Paris, France; 18 Service de médecine génétique, Hôpitaux Universitaires de Genève – HUG, Geneva, Switzerland; 19 Service de génétique médicale, Centre Hospitalier Universitaire Vaudois, Lausanne University, Lausanne, Switzerland; 20 Service de génétique médicale, CHU d’Angers, Angers, France; 21 Institut de Génétique Médicale, Hopital Jeanne de Flandre, Lille, France; 22 Hôpital Jeanne de Flandre, CHRU de Lille, Lille, France; 23 Genetica Medica, Dipartimento di Scienze Mediche, Università di Torino, Torino, Italy; 24 Département de génétique médicale, CHU de Marseille, Hôpital de la Timone, Marseille, France; 25 Centre de génétique humaine, CHU de Liège, Liège, Belgique; 26 Service de psychiatrie, Centre hospitalier de Rouvray, Sotteville lès Rouen, France; 27 Service de génétique médicale, Centre Hospitalier Bretagne Atlantique CH Chubert, Vannes, France; 28 Service de génétique clinique, CHU de Lyon, Hospices Civils de Lyon, Lyon, France; 29 Service de Génétique Médicale, CHU de Nantes, Hôtel Dieu, Paris, France; 30 Service de Génétique Humaine, CHU Sart Tilman, Liège, Belgique; 31 Service de génétique médicale, CHU de Bordeaux, Hôpital Pellegrin, Bordeaux, France; 32 Service de Génétique et Biologie de la Reproduction, CHU de Reims, Hôpital Maison Blanche, Reims, France; 33 Department of Pediatrics, Aabenraa Hospital, Sonderjylland, Denmark; 34 Service de génétique clinique, CHU de Lyon, Hospices Civils de Lyon, Lyon, France; 35 Department of Clinical Genetics, Odense University hospital, Odense, Denmark; 36 Centre de génétique, Hôpital d’Enfants, CHU Dijon Bourgogne - Hôpital François Mitterrand, Dijon, France; 37 Ambulatorio di Genetica Medica, Ospedali Galliera di Genova, Genoa, Italy; 38 Clinical Genetics Department, 7th Floor Borough Wing, Guy’s Hospital, Guy’s & St Thomas’ NHS Foundation Trust, Great Maze Pond, London, SE1 9RT UK; 39 Département de Génétique Médicale, Maladies Rares et Médecine Personnalisée, service de génétique clinique, Université Montpellier, Unité Inserm U1183, CHU Montpellier, Montpellier, France; 40 Service de Génétique, CHU de Caen, Hôpital Clémenceau, Caen, France; 41 Genetica Medica, Dipartimento di Scienze Cliniche e Biologiche, Università di Torino, Torino, Italy; 42 Service de génétique, CHU d’Angers, Angers, France; 43 Service de psychiatrie, Centre hospitalier du Rouvray, Sotteville lès Rouen, France; 44 Service de Génétique clinique, CHU Paris-GH La Pitié Salpêtrière-Charles Foix - Hôpital Pitié Salpêtrière, Paris, France; 45 Service de Génétique Médicale, CHU de Nantes, Hôtel Dieu, Paris, France; 46 Service de Génétique clinique, CHU Paris-GH La Pitié Salpêtrière-Charles Foix - Hôpital Pitié-Salpêtrière, Foix, France; 47 Service de Génétique Moléculaire et Génomique – Pôle biologie, CHU de Rennes, Hôpital Pontchaillou, Rennes, France; 48 Service de génétique médicale, Centre Hospitalier Bretagne Atlantique CH Chubert, Vannes, France; 49 Centre de Génétique Moléculaire et Chromosomique, CHU Paris-GH La Pitié Salpêtrière-Charles Foix - Hôpital Pitié-Salpêtrière, Paris, France; 50 Service de génétique médicale, CHU de Bordeaux-GH Pellegrin, Bordeaux, France; 51 Pediatric Neurology Unit, Department of Pediatrics, Lausanne University Hospital, Lausanne, Switzerland; 52 Service de Génétique Médicale - Institut de Biologie, CHU de Nantes, Nantes, France; 53 Service de Neuropédiatrie, Centre Hospitalier Régional Universitaire de Lille, Lille, France; 54 Service génétique médicale et oncogénétique, Hotel Dieu, Chambéry, France; 55 Service de Génétique Clinique, CHU Amiens Picardie, Amiens, France; 56 Service de Génétique Médicale, CHU de Nantes, Hôtel Dieu, Paris, France; 57 Service de Génétique clinique, CHU Paris-GH La Pitié Salpêtrière-Charles Foix - Hôpital Pitié-Salpêtrière, Paris, France; 58 Département de génétique médicale, CHU de Marseille, Hôpital de la Timone, Marseille, France; 59 Service de génétique clinique Guy Fontaine, Hôpital Jeanne de Flandre, CHRU de Lille, Lille, France; 60 Department of Clinical Genetics, Odense University Hospital, Odense, Denmark; 61 Département de Génétique Médicale, Maladies Rares et Médecine Personnalisée, service de génétique clinique, Université Montpellier, Unité Inserm U1183, CHU Montpellier, Montpellier, France; 62 Service de Génétique, CHU de Caen, Hôpital Clémenceau, Caen, France; 63 Service de génétique clinique, CHU de Saint-Etienne - Hôpital Nord, Saint-Priest-en-Jarez, France; 64 Center for Integrative Genomics, Lausanne University, Lausanne, Switzerland; 65 Laboratoire de génétique moléculaire, CHU de Bordeaux-GH Pellegrin, Bordeaux, France; 66 Service de génétique clinique, CHU de Lyon, Hospices Civils de Lyon, Lyon, France; 67 Laboratoire de Cytogénétique Constitutionnelle, CHU de Lyon, Hospices Civils de Lyon, Lyon, France; 68 Department of Clinical Genetics, Odense University Hospital, Odense, Denmark; 69 Laboratoire de Cytogénétique Constitutionnelle, CHU de Lyon, Hospices Civils de Lyon, Lyon, France; 70 Service de génétique clinique, CHU de Lyon, Hospices Civils de Lyon, Lyon, France; 71 Department of Genetics, University Medical Center Utrecht, Utrecht, Netherlands; 72 Centre de Génétique humaine, CHRU de Besançon - Hôpital Saint-Jacques, Besançon, France; 73 Simons Foundation, 160 Fifth Avenue, 7th Floor, New York, NY 10010 USA

**Keywords:** Clinical genetics, Neuroscience

## Abstract

Many copy number variants (CNVs) confer risk for the same range of neurodevelopmental symptoms and psychiatric conditions including autism and schizophrenia. Yet, to date neuroimaging studies have typically been carried out one mutation at a time, showing that CNVs have large effects on brain anatomy. Here, we aimed to characterize and quantify the distinct brain morphometry effects and latent dimensions across 8 neuropsychiatric CNVs. We analyzed T1-weighted MRI data from clinically and non-clinically ascertained CNV carriers (deletion/duplication) at the 1q21.1 (*n* = 39/28), 16p11.2 (*n* = 87/78), 22q11.2 (*n* = 75/30), and 15q11.2 (*n* = 72/76) loci as well as 1296 non-carriers (controls). Case-control contrasts of all examined genomic loci demonstrated effects on brain anatomy, with deletions and duplications showing mirror effects at the global and regional levels. Although CNVs mainly showed distinct brain patterns, principal component analysis (PCA) loaded subsets of CNVs on two latent brain dimensions, which explained 32 and 29% of the variance of the 8 Cohen’s d maps. The cingulate gyrus, insula, supplementary motor cortex, and cerebellum were identified by PCA and multi-view pattern learning as top regions contributing to latent dimension shared across subsets of CNVs. The large proportion of distinct CNV effects on brain morphology may explain the small neuroimaging effect sizes reported in polygenic psychiatric conditions. Nevertheless, latent gene brain morphology dimensions will help subgroup the rapidly expanding landscape of neuropsychiatric variants and dissect the heterogeneity of idiopathic conditions.

## Introduction

Genomic copy number variants (CNVs) are deletions or duplications of DNA segments of more than 1000 base pairs. Rare CNVs with large effects have been associated with a range of often overlapping developmental psychiatric phenotypes and conditions, including autism spectrum disorder (ASD) and schizophrenia (SZ) [1–4]. A looming question in psychiatric genetics pertains to the underlying basis of polygenicity: How do different variants lead to risk for the same psychiatric condition?

Some of the most frequent risk factors for neuropsychiatric disorders identified in pediatric clinics include CNVs at the 22q11.2, 16p11.2, 1q21.1, and 15q11.2 genomic loci [5, 6]. They affect the dosage of 60, 29, 12 and 4 genes, respectively [7–9]. The largest increases in risk for SZ have been documented for the 22q11.2 deletion (30 to 40-fold) followed by 16p11.2 duplication (10-fold), 1q21.1 deletion and 15q11.2 deletion (1.5-fold) [2]. ASD risk is highest for 16p11.2 deletions and duplications (10-fold) followed by 1q21.1 duplications and 22q11.2 duplications (3 to 4-fold) [1, 2, 10–13]. The nature and specificity of CNV effects on cognitive and behavioral traits is an area of intense investigation. All CNVs studied to date affect cognition to varying degrees and a broad range of cognitive functions [14, 15]. A recent study found that the range of affected traits was broadly similar for 13 CNVs at 8 loci and specific genotypes accounted for a low proportion of phenotypic variance [3]. These variants are therefore opportunities to investigate brain phenotypes conferring high-risk for mental illness.

Neuroimaging studies have only been performed for a few CNVs. Robust effects on total and regional brain volumes, cortical thickness (CT) and surface area (SA), have been reported in 22q11.2 [12, 13, 16], 16p11.2 BP4-5 [17–19], and 15q11.2 CNVs [20–23]. Opposing effects on global and-or regional brain volumes between deletions and duplications were observed for 16p11.2 [19], 22q11.2 [16], 1q21.1 [24] and 15q11.2 [20] loci (hereafter referred to as “mirror effects”).

Neuroanatomical alterations associated with 16p11.2 and 22q11.2 show overlap with those observed in idiopathic ASD and SZ [17–19, 21, 25]. Finally, most of the effects are observed irrespective of psychiatric diagnoses and symptoms [12], suggesting that the final clinical outcome may result from the effect of CNVs and additional factors.

Neuroimaging studies across genomic variants are scarce. An investigation of 49 unaffected carriers of SZ-associated CNVs across five genomic loci in the UK biobank showed smaller volumes of the thalamus, hippocampus, and nucleus accumbens [26]. Functional connectivity similarities have also been demonstrated between 16p11.2 and 22q11.2 deletions as well as with idiopathic ASD and SZ [27]. Alternatively, a recent study suggests a relatively distinct association between neuroimaging alterations and six different CNVs [28].

In this study, we aimed to characterize shared and distinct neuroanatomical alterations associated with eight CNVs at four genomic loci. We analyzed high-resolution structural brain scans from the largest multi-site dataset of CNV carriers (*n* = 484, of which 87 have not yet been published) and controls (*n* = 1296) to date. Different approaches were implemented, from simple case-control contrasts to one-view and multi-view multivariate pattern learning [29, 30]. First, we compared brain morphometry features associated with each deletion and duplication using univariate linear models. Second, we quantified the shared variation of brain morphometry associated with eight CNVs using principal component analysis (PCA). To complement this single-view approach, a multi-view pattern-learning algorithm was carried out for the joint analysis of genetic and morphometry brain data, to identify latent ‘gene-morphometry dimensions’ (canonical correlation analysis, CCA). Primary analyses were performed using VBM for consistency with previous studies [19]. In addition, we carried out the same multivariate analyses using freesurfer-derived cortical SA and thickness to ensure that shared variation was not limited to one neuroimaging modality or analytical pipeline.

## Methods

### Participants

Deletions and duplications carriers’ neuroimaging data included in the study were selected on the following breakpoints (hg 19): 16p11.2 (BP4-5, 29.6-30.2MB), 1q21.1 (Class I, 146.4-147.5MB & II, 145.3-147.5MB), 22q11.2 (BPA-D, 18.8-21.7MB) and 15q11.2 (BP1-2, 22.8–23.0MB), together with control individuals not carrying any CNVs at these loci (Table 1, Supplementary Table 1 and supplementary materials). Signed consents were obtained from all participants or legal representatives prior to the investigation. Of note, data of 87 CNV carriers have never been published. Clinically ascertained CNV carriers were recruited as either probands referred for genetic testing, or as relatives. Controls were either non-carriers within the same families or individuals from the general population. We pooled data from five cohorts. CNVs from non-clinical populations were identified in the UK Biobank [31, 32].

**Table 1:** Demographics.

**Clinical ascertainment**								
**CNV loci**	**Copy number**	**Age mean (SD)**	**Male/Female**	**TIV mean (SD)**	**FSIQ mean (SD)**	**ASD**	**SCZ**	**Other diagnosis**
1q21.1	Deletions *N* = 29	29 (18)	11/18	1.22 (0.14)	90.85 (21.75) *N* = 26	1	–	7
	Duplication *N* = 19	34 (17)	10/9	1.57 (0.11)	95.56 (23.19) *N* = 18	1	–	4
16p11.2	Deletions *N* = 83	17 (12)	47/36	1.54 (0.17)	82.17 (14.99 *N* = 64	13	–	36
	Duplication *N* = 73	31 (14.9)	41/32	1.33 (0.17)	85.47 (19.48) *N* = 63	10	1	19
22q11.2	Deletions *N* = 74	16 (8.6)	35/39	1.30 (0.15)	77.42 (13.51) *N* = 48	9	2	32
	Duplication *N* = 22	20 (14.2)	15/7	1.47 (0.16)	97.83 (20.34) *N* = 12	2	–	8
Controls *N* = 331		26 (14.6)	189/142	1.46 (0.15)	106.73 (15.03) *N* = 224	1	–	23
Non-clinical ascertainment								
**CNV loci**	**Copy number**	**Age mean (SD)**	**Male/Female**	**TIV mean (SD)**	**UKB FI mean (SD)**	**ASD**	**SCZ**	**Other diagnosis**
1q21.1	Deletions *N* = 10	59.1 (6.7)	6/4	1.35(0.12)	−0.8 (0.5) *N* = 9	–	1*	3
	Duplication *N* = 9	60.6 (7)	2/7	1.55(0.14)	0.2 (1.3) *N* = 9	–	–	–
15q11.2	Deletions *N* = 72	63.4 (7.6)	31/41	1.54(0.15)	−0.3 (0.9) *N* = 63	–	–	2
	Duplication *N* = 76	62.9 (7.3)	36/40	1.49(0.15)	0 (1.1) *N* = 71	–	–	6
16p11.2	Deletion *N* = 4	65.6 (3.2)	3/1	1.56(0.13)	0.8 (0.5) *N* = 2	–	–	–
	Duplication *N* = 4	69.3 (2.1)	1/3	1.29(0.11)	−1.6 (0.2) *N* = 4	–	–	–
22q11.2	Deletion *N* = 1	69.8 (–)	1/–	1.44(-)	–	–	–	–
	Duplication *N* = 8	62 (9.5)	4/4	1.55(0.17)	−0.2 (1.1) *N* = 8	–	–	1
Controls N = 965		62.1 (7.4)	358/607	1.51(0.14)	0 (1) *N* = 866	–	2*	65

*CNV* copy number variant, *SD* standard deviation, *TIV* total intracranial volume, *FSIQ* full scale IQ, *UKB FI* UK Biobank fluid intelligence, *ASD* autism spectrum disorders, *SCZ* schizophrenia (including * ICD10 code F25.9 Schizoaffective disorder, unspecified).

CNV carriers and controls from the clinically ascertained group come from five different cohorts (Supplementary Table 1), while non-clinically ascertained participants were identified in the UK Biobank. 16p11.2 and 22q11.2 from the UKBB were not included in the VBM and SBM due to small sample size. Other diagnosis included: language disorder, major depressive disorder, posttraumatic stress disorder (PTSD), unspecified disruptive and impulse-control and conduct disorder, social anxiety disorder, social phobia disorder, speech sound disorder, moderate intellectual disability, specific learning disorder, gambling disorder, bipolar disorder, conduct disorder, attention deficit/hyperactivity disorder ADHD, Substance abuse disorder, global developmental delay, motor disorder, obsessive compulsive disorder, sleep disorder, Tourette’s disorder, mood disorder, eating disorders, transient tic disorder, trichotillomania, pervasive developmental disorder NOS, specific phobia, body dysmorphic disorder, mathematics disorder, dysthymic disorder.

### MRI data

Details for methods and analyses are provided in Supplementary material and Supplementary Methods 1–8. Data sample included T1-weighted (T1w) images at 0.8–1 mm isotropic resolution across all sites. Population description is available in Table 1 and Supplementary Table 1.

### Data quality check

All data included in the analysis were quality checked by the same researcher (CM). A total of 107 structural brain scans from carriers and controls were excluded from further analysis based on visual inspection that identified significant artifacts compromising accurate tissue classification and boundary detection (Supplementary materials).

### MRI data processing

Data for Voxel-Based Morphometry were preprocessed and analyzed with SPM12 (http://www.fil.ion.ucl.ac.uk/spm/software/spm12/) [33–35] running under MATLAB R2018b (https://www.mathworks.com/products/new_products/release2018b.html). For surface-based feature extraction, we used FreeSurfer 5.3.0 (http://surfer.nmr.mgh.harvard.edu [36,37,]). Quality control was performed using standardized ENIGMA quality control procedures (http://enigma.ini.usc.edu/protocols/imaging-protocols/).

### Statistical analysis for global brain measures

Global brain aggregate measures (TIV, total gray matter (GM) volume, total SA, and mean CT) were adjusted for age, age^2^, and sex as fixed effects and scanning site as random factor. Non-clinically ascertained subjects from the UKBB are on average 30 years older than the clinically ascertained subjects. Because of this age difference we used age matched control groups for univariate analysis. Global measure z-scores for each CNV for clinically and non-clinically ascertained CNVs were calculated using 331 and 965 controls, respectively. All statistical analyses were performed in R, version 3.4.4 (https://www.r-project.org/), or in MatlabR2018b.

### Voxel-based measures and statistical analyses

We performed whole-brain voxel-based analysis testing for voxel-wise volume differences within the mass-univariate analysis framework implemented in SPM (Supplementary Method 4). Cohen’s *d* (i.e., effect size) [38] maps were obtained by converting SPM T-maps using the CAT12 toolbox for SPM (http://www.neuro.uni-jena.de/cat/).

### Surface-based measures and statistical analyses

In parallel to VBM, we used surface-based GLM-based analysis to test differences in CT and SA (SurfStat toolbox [39]).

### Neuromorphometrics and Desikan parcellations

Parcellation into regions of interest (ROIs) was performed using neuromorphometric atlas (http://www.neuromorphometrics.com/) for GM volume (130 ROIs excluding white matter ROIs), and using Desikan parcellation [37] for FreeSurfer-derived CT and SA (68 ROIs).

### Comparison of ranked Cohen’s *d* maps across CNVs

To adjust for the unequal power to detect alterations across different CNV groups, which have different sample and effect sizes, we ranked the Cohen’s *d* values of all voxels (/vertices) for each statistical maps (CNV versus controls contrast). We then tested for spatial overlap between maps across CNVs after thresholding the tails of the distribution at the 15th & 85th quantiles. Dice index was calculated using publicly available Matlab scripts (https://github.com/rordenlab/spmScripts).

### Null hypothesis testing using spin permutations and label shuffling

We used spin permutation and label shuffling [40, 41] to calculate empirical *p* values for (1) the deletion and duplication convergence pattern and (2) the correlation/dice index between two maps.

### Quantifying shared variation across CNVs using principal components (PC)

PCs were derived to quantify shared morphometry variation across CNVs. We used Cohen’s *d* values of 130 neuroanatomical GM regions (neuromorphometrics atlas) of eight CNVs as input-variables (*z*-scored Cohen’s *d* contrasts adjusted for total GM and nuisance variables; FactoMineR package in R). The variance explained (coefficient of determination, R-squared) for each CNV-associated Cohen’s d map by PCs was obtained by running a linear model (lm) in R; with PC1 and PC2 as independent explanatory variables and the CNV Cohen’s d map as a dependent variable.

### Jointly modeling of gene-morphology dimensions using CCA

We re-purposed CCA to simultaneously model the shared and distinct impact of the CNVs in causing distributed alterations in brain morphometry (130 grey matter regions) [29, 30]. This principled doubly-multivariate approach, widely used in neuroimaging studies [29, 30], was performed to identify modes of coherent co-variation that jointly characterize how CNVs and patterns of regional volumes systematically co-occur across subjects. Henceforth, we refer to the ensuing modes of co-variation as ‘CCA dimensions’ or ‘gene-morphology dimensions’.

## Results

### CNV effects on global brain morphometry

**Fig. 1:**
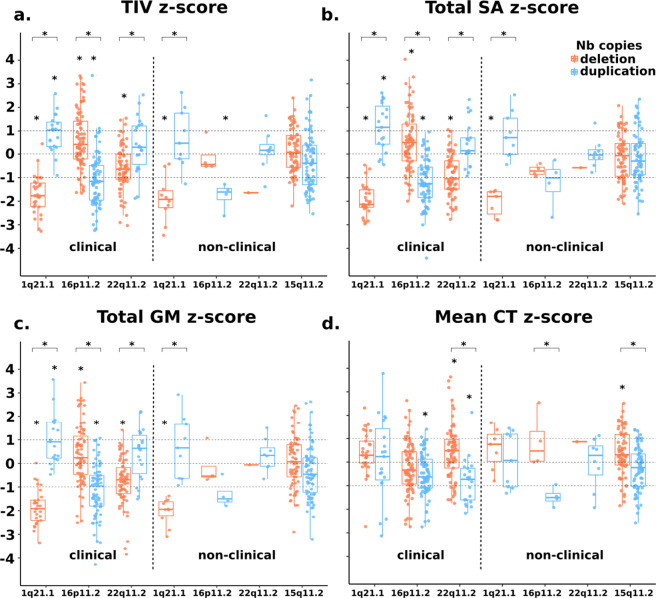
1q21.1, 16p11.2, 22q11.2, and 15q11.2 exert rich effects on global brain measures. Total intracranial volume (**a**), total surface area (**b**), total grey matter volume (**c**) and mean cortical thickness (**d**) for clinically and non-clinically ascertained CNVs. *Z* scores for clinically and non-clinically ascertained CNVs were calculated using 331 and 965 controls, respectively, adjusting for age, age^2^, sex and site as a random factor. *Y* axis values are *z* scores. *X* axis are CNV groups. Significant difference between CNV group and corresponding control group is indicated with a star. Horizontal bars with stars show significant differences between deletions and duplications within the same locus. TIV total intracranial volume, SA surface area, GM grey matter, CT cortical thickness.

Deletions and duplications of each genomic loci showed opposing effects on one or more global metrics: TIV, total GM volume, total SA, or mean CT (Fig. 1, Supplementary Table 2). The directionality of global effects differed across loci (Fig. 1a–c). Effects on GM and SA were less pronounced once adjusted for TIV (Supplementary Fig. 1).

### Overlapping deletion effects on regional morphometry

**Fig. 2:**
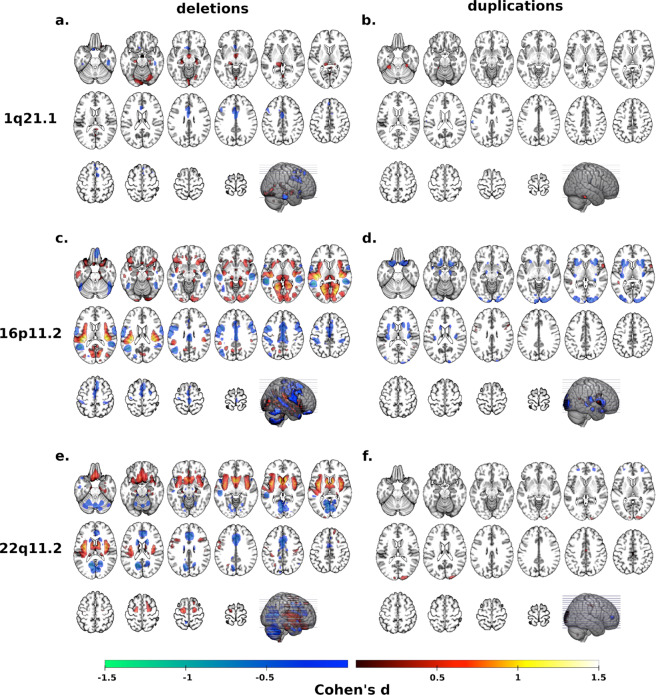
Cohen’s *d* maps of VBM regional brain differences in deletion and duplication carriers at the 1q21.1, 16p11.2, and 22q11.2 loci compared to controls. Regional brain differences adjusted for total grey matter volume. Left and right columns show results for deletions (**a**, **c**, **e**) and duplication (**b**, **d**, **f**) carriers, respectively. Color maps show the significant effects of each CNV, thresholded at *q* < 0.05 FWE. Color scale represents positive and negative Cohen’s *d* effect sizes were estimated. The linear model was adjusted for sex, linear, and quadratic expansion of age and total grey matter volume. 15q11.2 was not displayed because only a few voxels survived family-wise error (FWE) correction. Corresponding maps for surface area and cortical thickness are reported in Supplementary Figs. 4 and 5.

**Fig. 3:**
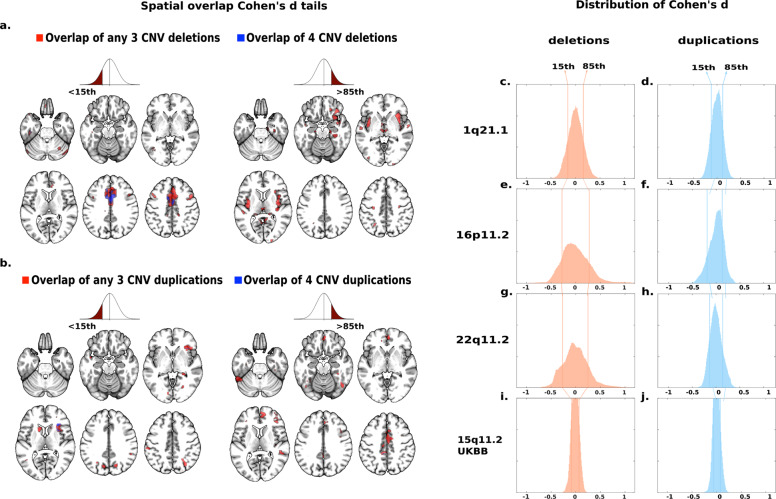
Spatial overlap across deletions and duplications at four genomic loci. Spatial overlap across clinically and non-clinically ascertained deletions (**a**) and duplications (**b**) at four genomic loci shown separately for <15th and >85th percentile of Cohen’s *d* values. Overlap of all four deletions (**a**) or all four duplications (**b**) is shown in blue. Overlaps of any combination of three deletions (**a**) or any combination of three duplications (**b**) are shown in red. Top ranking Cohen’s d values used in (**a**, **b**) are presented on the density plots for all eight deletions and duplications: 1q21.1 (**c**, **d**), 16p11.2 (**e**, **f**), 22q11.2 (**g**, **h**), 15q11.2 (**i**, **j**). The *x axes* values of the eight density plots are Cohen’s *d*. Corresponding maps for surface area and cortical thickness are reported in Supplementary Figs. 6 and 7.

Whole-brain VBM analyses contrasting each deletion and duplication group with controls showed mostly distinct brain patterns across CNVs (Fig. 2a, c, e, Supplementary Table 3). To investigate potential overlap across the four genomic regions, we ranked Cohen’s *d* maps and overlapped voxels with similar rankings. Using a threshold for voxels with Cohen’s *d* < 15th and >85th percentiles separately (Fig. 3c, e, g, i), we observed significant overlap between deletions (*p* value_SHUFFLE_ < 10e−4, Fig. 3a). Volumes of the middle and anterior cingulate extending to the supplementary motor cortex and of the cerebellum were decreased in all deletions while volume was increased in the thalamus (Fig. 3a).

**Fig. 4:**
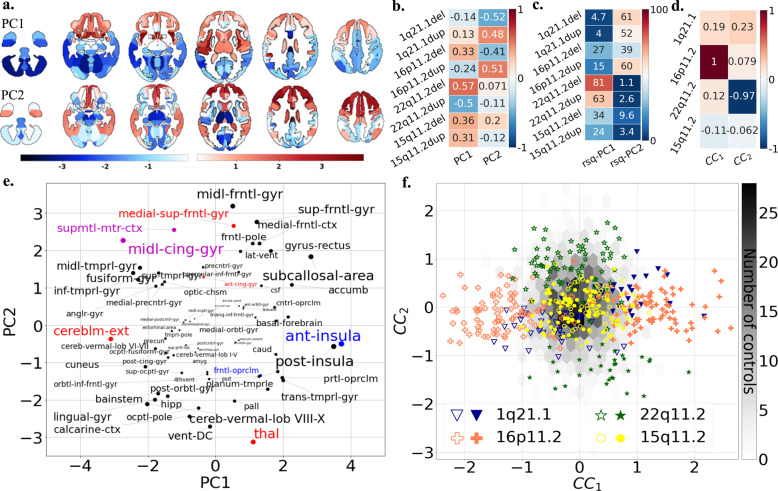
Principal component analysis and canonical correlation analysis of brain alterations due to eight CNVs. **a** PCA dimension 1 and 2 regional relevances projected on axial brain slices. The darker the red or blue color, the stronger the positive or negative association with the PCA dimensions. PCA was run on *z*-scored Cohen’s *d* values, with the eight CNVs as variables and 130 neuroanatomical GM regions as observations. GM region volumes were adjusted for total grey matter, age, age^2^, sex, and site. The first two components explained respectively 31.77 and 28.66 % of the variance. **b** Loading of eight CNVs on the two PCA dimensions. Values are PC loading magnitudes and represent the contribution of a CNV to the PC. **c** Variance explained (coefficient of determination, R-squared) of each CNV Cohen’s *d* profile by PC1 and PC2. Values and color scale represent the “percent of variance”. **d** Loadings of the first and second CCA dimension on four CNV genomic loci. Shows contribution of a CNV loci to the canonical dimension. **e** Loading of Neuromorphometrics Regions of Interests (ROIs) on the two PCA dimensions. ROIs are averaged across the left and right hemisphere for visualization. The font size is correlated to the region’s contribution to dimensions. ROI names are color coded as being part of the deletion (red), duplication (blue) and both deletion and duplication (magenta) convergence patterns. **f** Scatterplot showing the participant/specific expressions of each of the 484 carriers of eight different CNVs along two dominant gene-morphometry canonical correlation (CC) dimensions established using 130 neuroanatomical GM regions of CNV carriers. GM region volumes were adjusted for total grey matter, age, age^2^, sex, and site. The empty and full symbols represent deletions and duplication, respectively. The grey hexagonal bin plot represents the frequency of controls (*n* = 1296). Controls were not used to calculate the CCA and were projected post hoc on the two dimensions using CCA prediction. CCA ROI loadings are reported in Supplementary Fig. 10. Results for surface area and cortical thickness are reported in Supplementary Fig. 9 (PCA), 14–15 (CCA).

Sensitivity analyses tested the effect of ascertainment and control groups: (1) We recomputed the deletion convergence map using 1q21.1 deletion carriers from UK Biobank instead of those clinically ascertained (Table 1). The new deletion convergence map was similar to the initial one presented above with a dice index of 39.4% (*p* value_SPIN_ < 10e−4); (2) We excluded all subjects with autism, SZ, or other psychiatric diagnoses. Again, this did not change the overlap (Supplementary Fig. 2); (3) We tested the effects of the control group by recomputing contrasts only using controls from the same site (instead of the initial ANOVA pooling all controls together and controlling for site). This again did not alter the convergence maps (Supplementary Fig. 3). Finally, we performed the same analysis using Freesurfer-derived SA and CT measures. We also identified spatial overlaps but regions identified were different especially for CT (Supplementary Table 4 & Fig. 4). Overlap maps are provided in Supplementary Figs. 5–8 and Tables 5, 6.

### Overlapping duplication effects on regional morphometry

Contrasts computed for duplications (Fig. 2b, d, f) showed smaller effect sizes compared to deletions. The same analysis using Cohen’s *d* values <15th and >85th percentiles (Fig. 3d, f, h, j) demonstrated spatial overlap across all four duplications (*p* value_SHUFFLE_ < 10e−4, Fig. 3b). The resulting pattern was mainly distinct from the one observed in deletions and was characterized by smaller volumes in anterior insula and frontal operculum, and larger volumes in the middle cingulate gyrus and supplementary motor cortex compared to controls.

Sensitivity analysis testing the effect of clinical ascertainment, psychiatric diagnoses, control groups, and volume versus Freesurfer-derived measures demonstrated that results were robust (Supplementary Figs. 2–8).

The deletion/duplication ratio of Cohen’s *d* distributions ranged from 1.24 to twofold across the four genomic loci (*F*-test, *p* < 10e^−16^, Fig. 3c–j, Supplementary Table 7). Similar effect-size ratios were also observed for SA alterations (Supplementary Table 7), except for the 15q11.2 locus.

We tested opposing (mirror) effects on VBM contrast maps between deletion and duplications. The strongest anticorrelation of Cohen’s *d* values was observed for 16p11.2 (*p* value_SPIN_ < 10e−4) followed by 15q11.2 (*p* value_SPIN_ < 10e−4), 1q21.1 (*p* value_SPIN_ < 0.033) and 22q11.2 (*p* value_SPIN_ < 0.038) (Supplementary Fig. 9 and Tables 8–10). Mirror effects were observed in clinically and non-clinically ascertained CNV carriers, as well as for SA at all four genomic loci but not for CT (Supplementary Tables 8–10). Hence mirror effects were observed in global metrics and, independently, in regional alterations.

### Quantifying distinct and shared effects on brain morphometry associated with eight CNVs

We performed a multivariate PCA based on Cohen’s *d* profiles obtained from contrasts between the eight CNV groups and controls (using 130 neuromorphometric regional volumes, Supplementary Table 11). The first two components explained 31.8 and 28.7% of the variance of Cohen’s *d* maps, respectively. The third component dropped to 13.8% and was therefore not investigated further.

Deletions and duplications at each genomic loci showed opposite loading on PC1 or PC2 (Fig. 4c). Regions with the highest loadings on PC1 and PC2 were also those identified in the convergence maps presented above: in particular the middle cingulate gyrus and the supplementary motor cortex. Anterior and posterior insula, cerebellum, fusiform gyrus and thalamus were also top regions altered across subsets of CNVs (Fig. 4a, b and Supplementary Table 12). The variance explained by both components for each CNV’s Cohen’s *d* map ranged from 27% to 82% (Fig. 4d). Finally, we performed the same analysis using Freesurfer-derived SA and CT measures which also provided latent dimensions with comparable variance explained, opposing loadings for deletions and duplications of each genomic loci (Supplementary Fig. 10). However, CNV loadings differ across brain morphometry metrics.

### Gene-morphology dimensions across eight CNVs

As a next step, we performed a multi-view pattern-learning analysis, jointly analyzing the genetic and morphometry brain data. This doubly multivariate method allowed testing whether shared dimensions could be identified in a data-driven approach, without performing any individual contrast. We interrogated 2 hypotheses: (1) CNVs show levels of shared brain effects at the morphometry level and (2) deletions and duplications show opposing effects. We investigated the same 130 regional volumes in 484 carriers of CNVs at four genomic loci. To test hypothesis (2), deletions and duplications were coded as opposing gene dosage. CCA confirmed both hypotheses by identifying two significant ‘gene-morphometry dimensions’ (*r* = 0.84, 0.79, *p* value < 0.05, Fig. 4e, f). Regional brain contributions to canonical dimension 1 and 2 were well correlated with those of PC2 and 1, respectively (*r* = 0.83, *r* = −0.81).

Top ranking brain regions contributing to either of the two CCA dimensions of morphological variation included supplementary motor cortex, posterior and anterior insula, middle cingulate gyrus, calcarine cortex, cuneus and accumbens (Supplementary Fig. 11 and Supplementary Table 13). 16p11.2 and 22q11.2 preferentially contributed to dimension 1 and 2 respectively, and 1q21.1 loaded similarly on both dimensions. 15q11.2 CNVs showed the smallest loadings on both dimensions (Fig. 4e).

Sensitivity analyses are detailed in supplementary material (Supplementary Figs. 12–16 and Tables 14, 15).

## Discussion

Here, in the largest cross-CNV-neuroimaging study to date, we tested potentially shared effects of eight neuropsychiatric CNVs on brain morphometry. CNVs showed a combination of distinct and shared profiles of brain alterations, as demonstrated by the spatial overlap of Cohen’s *d* maps across deletions and duplications. A multivariate approach (PCA) quantified distinct and shared alterations across subsets of CNVs and identified two latent dimensions explaining 31.8 and 28.7% of Cohen’s *d* map’s variance. A second multivariate approach (CCA), jointly analyzing genetic and morphometry data, confirmed the latent CNV-brain dimensions identified by PCA. Genomic loci contributed to the latent CCA dimensions in proportion to their effect sizes. Even for small effect-size deletions at the 1q21.1 and 15q11.2 loci, the PCA components explained between 43 and 65% of their Cohen’s *d* profile. All three approaches—spatial overlap, CCA, and PCA—identified a similar set of regions altered by CNVs including the cingulate gyrus and supplementary motor cortex.

### Distinct and shared effects of CNVs

Our results show that two-thirds of the average CNV effects on brain morphometry are distinct. This is consistent with a recent study showing relative specificity of association between brain patterns of gene expression and patterns of cortical anatomy changes across six CNVs and chromosomal aneuploidies [28]. One-third of the effects on brain morphometry is shared as demonstrated by latent gene-morphology dimensions identified across subsets of CNVs. There is no single dimension explaining CNV effects. Instead, subsets of CNVs load on either dimension, which may suggest similar brain mechanisms within subgroups of CNV. Yet CNVs within subgroups were not characterized by the same risk for ASD or SCZ.

These results have implications for our conceptualization of polygenic psychiatric conditions. Indeed, studies estimate that 70–100% of any 1-MB window in the human genome encompasses variants (including CNVs) contributing to increased risk for SZ and autism [4, 42]. Gene-morphology dimensions alone, can not explain the fact that subgroups of CNVs are associated with a similar range of behavioral symptoms [43], and psychiatric disorders [1, 2, 4, 44]. In fact, the large proportion of distinct CNV-neuroimaging effects suggests that a broad diversity of brain mechanisms increase the risk for autism and SZ. Extreme examples include CNVs associated with opposing loadings on the same latent gene-morphology dimension while increasing risk for the same psychiatric condition (ie. 16p11.2 deletions, duplications, and autism). The presence of such genomic variants in studies of ASD and SZ may explain heterogeneity and small neuroimaging effect sizes [45, 46]. Why opposing effects on the same latent brain dimension increase risk for the same psychiatric condition is an unsolved question. Further observations on a broad variety of genomic variants are required to address this question.

### Brain hubs vulnerable to altered gene dosage

Insula, cingulate, fusiform gyrus, and hippocampus are regions showing alterations across SZ, bipolar disorders, major depression, and obsessive-compulsive disorders [45, 47]. The cingulate, insula, and fusiform gyrus were also among regions markedly altered across eight CNVs. CNVs have either negative or positive effects on these brain regions, however, the number of CNVs included in this study did not allow us to associate the directionality of these effects with phenotypic traits. Alterations of the cingulate cortex have been associated with genetic and environmental risk for SZ [48]. The supplementary motor cortex has been shown to play a critical role in 16p11.2, 22q11.2 CNVs as well as autism and SZ by functional connectivity studies, but not by cross-diagnostic neuroimaging structural studies [49, 50]. Several cerebellar regions (vermis lobule VIII-X and cerebellar cortex) are highly sensitive to CNVs, which may be due to the cerebellum’s protracted development [51]. The cerebellum has either been excluded or not reported by cross-disorder structural neuroimaging studies, but volume alterations have been associated with autism and SZ separately [52, 53]. Multiple genetic mouse models of autism, as well as Down Syndrome, also show abnormal cerebellar development [54]. The same level of spatial overlap was observed for SA and CT but implicated mostly distinct sets of brain regions. This is in line with the distinct genetic contributions previously demonstrated for these cortical metrics [55].

### Dissociation between global and regional effects

Results suggest that global and local effects may be mechanistically unrelated. 1q21.1 deletions and duplications highlight the contrast between very large effects on global measures, with small regional effects once adjusted for total GM. Dissociation is also observed between the directionalities of global and regional effects: all deletions are associated with a smaller cingulate and supplementary motor cortex volume irrespective of their effect on TIV and GM. Animal studies have proposed mechanisms for global [8, 56], but not regional effects of CNVs.

### Limitations

Multiple sites included in the study may have introduced noise, but previous studies have shown that site effects do not influence the neuroanatomical patterns associated with CNVs at the 16p11.2, 22q11.2, and 15q11.2 loci [12, 19, 23]. While shared variation could have been influenced by clinical ascertainment or psychiatric diagnoses, our sensitivity analyses showed that this is not the case. The effect of medication on CNVs brain alterations could not be investigated in the current study as medication information was not available for the whole dataset. We were underpowered to properly investigate potential sex-related effects of 1q21.1 and 15q11.2 on brain morphometry. Of note, previous neuroimaging studies of large 22q11.2 and 16p11.2 samples were unable to identify any sex-related effects [19, 25].

15q11.2 deletions and duplications have small effect sizes and larger samples would improve the accuracy of the brain morphometry signature. Systematic analysis through the two most widespread computational neuroanatomy frameworks (voxel-based and surface-based) shows that effects could not be attributed to the processing pipeline. Extending our approach to the rapidly expanding number of rare genomic variants associated with psychiatric disorders is required to draw a robust conclusion on the distinct and shared effects of CNVs on brain structure.

## Conclusions

The simultaneous analyses and comparisons of several genomic variants demonstrate distinct CNV-associated alteration profiles as well as shared latent gene-morphology dimensions relevant to subsets of CNVs. Large proportions of distinct effects may provide some answers to the small neuroimaging effect sizes reported in idiopathic psychiatric conditions. The mechanisms underlying the identified latent dimensions remain unknown and pathway convergence may occur early on at the transcriptome and protein level, or at later stages (i.e., brain architecture or behavior). The hotly debated omnigenic model postulates that convergence may occur at early stages due to highly interconnected cell regulatory networks [57]. These approaches may help subgroup genomic variants based on their morphometry signature and dissect the heterogeneity of psychiatric conditions.

## Supplementary information

Supplementary material
