# Supplementary material for: Effects of eight neuropsychiatric copy number variants on human brain structure
Source: Transl Psychiatry. 2021 Jul 20;11:399. doi: 10.1038/s41398-021-01490-9 (PMC8292542; doi:10.1038/s41398-021-01490-9)
Supplement: Supplementary file 1 — Supplementary material [file 41398_2021_1490_MOESM1_ESM.docx]

# **Supplementary Material, Methods and Results**

**INDEX**

[**Supplementary Material, Methods and Results**](#_fnk6b7di15im) 1

[MRI data acquisition and quality check](#_5nt7kfj2pena) 3

[Participants](#_hzjvin6w9a85) 3

[16p11.2 European Consortium](#_6e1o9r36jvb) 3

[Simons Searchlight Consortium](#_67alnxuw4x6o) 4

[Brain Canada](#_dbceu9awxq4v) 4

[UCLA](#_45pkitcvnixw) 5

[Cardiff](#_lh3zw67dfmvk) 5

[MRI quality control](#_zel8dnqae3b) 6

[List of abbreviations](#_hwerdhtpyph5) 6

[Supplementary Method 1: MRI data processing, Voxel-Based Morphometry](#_xjdwn92cq39p) 6

[Supplementary Method 2: MRI data processing, Surface-Based Morphometry](#_8gr3fncnq781) 7

[Supplementary Method 3: Statistical analysis for global brain measures](#_iazqmzwk8a9f) 7

[Supplementary Method 4: Voxel-based measures and statistical analyses](#_wuvdej74qunm) 7

[Supplementary Method 5: Surface-based measures and statistical analyses](#_v8qq0d1to142) 8

[Supplementary Method 6: Spin permutation testing](#_ere4rkz29gf1) 8

[Supplementary Method 7: Label shuffling](#_1rmjn146gyh2) 9

[Supplementary Method 8: Multi-view pattern-learning analysis](#_2srqghe0ohqu) 9

[Supplementary Table 1 : Demographics](#_7cqrkadi9yr) 11

[Supplementary Table 2: Mean and standard deviation for each CNV on global metrics.](#_uonc4sp30vhc) 13

[Supplementary Table 3: CNV effect sizes on local grey matter volume changes](#_s3gsycjhk46o) 14

[Supplementary Table 4: Intersection of overlap maps across SA-CT-VBM](#_sgeitk5a41ki) 16

[Supplementary Table 5: CNV effect sizes on local Surface Area changes](#_unzihwdvib4t) 17

[Supplementary Table 6: CNV effect sizes on local Cortical Thickness changes](#_lxavvqggdcpy) 18

[Supplementary Table 7: Within loci comparison of Cohen’s d variances between deletions and duplications](#_ulwoerin7tmv) 19

[Supplementary Table 8: Brain-wide Cohen’s d map Correlations for VBM](#_e30pxky19y5g) 20

[Supplementary Table 9: Cortex-wide Cohen’s d map Correlations for SA](#_eurq29ck4qng) 21

[Supplementary Table 10: Cortex-wide Cohen’s d map Correlations for CT](#_udgivlliyhvn) 21

[Supplementary Table 11: Cohen’s d values for neuromorphometric brain regions.](#_hctkcp8gjeti) 26

[Supplementary Table 12: Principal Component Analysis ROIs loadings (volume)](#_fd8ckg2s93fe) 27

[Supplementary Table 13: Canonical Correlation Analysis ROIs loadings (volume)](#_26nz19l4unp2) 28

[Supplementary Table 14: Canonical Correlation Analysis ROIs loadings (CT)](#_kjzhl5vk7sdh) 29

[Supplementary Table 15: Canonical Correlation Analysis ROIs loadings (SA)](#_wwv2f1j2oy30) 30

[Supplementary Figure 1: The effect of 1q21.1, 16p11.2, 22q11.2 and 15q11.2 on total GM and total SA adjusted for TIV](#_v98ufxgm71my) 31

[Supplementary Figure 2: Robustness of spatial overlap after removing subjects with psychiatric diagnosis](#_ub1alwju2c8x) 32

[Supplementary Figure 3: Robustness of spatial overlap after matching controls by site](#_l1qokn7id46w) 33

[Supplementary Figure 4: Intersection of deletion and duplication overlap maps of 4 CNVs across SA-CT-VBM](#_x81t0a48smi8) 34

[Supplementary Figure 5: FDR corrected Cohen’s d maps of Surface Area](#_i2s9nkf49ad9) 35

[Supplementary Figure 6: FDR corrected Cohen’s d maps of Cortical Thickness](#_eb7ez093vise) 37

[Supplementary Figure 7: Spatial overlap for surface area (SA) across deletions and duplications](#_nve1lo2w9n0) 39

[Supplementary Figure 8: Spatial overlap for mean cortical thickness (CT) across deletions](#_k4oh56ulox38) 40

[Supplementary Figure 9: Cortex-wide mirror effects between deletions and duplications](#_773seb4nbg2r) 41

[Supplementary Figure 10: Principal Component Analysis of brain alterations associated with 8 CNVs for SA and CT](#_vop9o08pdo4p) 43

[Supplementary Figure 11: Co-analysis of shared brain alterations due to 8 CNVs (4 Genomic Loci)](#_11tf84bdiqu5) 45

[Supplementary Figure 12: CCA analysis of 8 CNVs (Deletion and Duplication as independent groups)](#_ollh411q7e2h) 47

[Supplementary Figure 13: Impact of each genomic loci on Canonical Correlations](#_3vwtyqopz9ln) 48

[Supplementary Figure 14: Impact of sample size on CCA analysis](#_o4bl25ihes3f) 49

[Supplementary Figure 15: CCA analysis across 4 Genomic Loci, Cortical Thickness](#_o1yqv4ku3koe) 50

[Supplementary Figure 16: CCA analysis across 4 Genomic Loci, Surface Area](#_qz2pt7hxrx03) 52

[References](#_myxnqid8jrn9) 54

## MRI data acquisition and quality check

#### **Participants**

Clinically ascertained CNV carriers were recruited as either probands referred for genetic testing, or as relatives. Controls were either non-carriers within the same families or individuals from the general population. We pooled data from 5 different cohorts: Cardiff University (UK), 16p11.2 European Consortium (Lausanne, Switzerland), University of Montreal (Canada), UCLA (Los Angeles, USA) and the Variation in individuals Project (SVIP, USA). A subset of the participants with 16p11.2 and 22q11.2 CNVs were included in prior publications [^1–4^](https://www.zotero.org/google-docs/?5cUR0z) (Supplementary Table 1). CNVs from non-clinical populations were identified in the UK Biobank [^5,6^](https://www.zotero.org/google-docs/?MRwuvE). PennCNV and QuantiSNP were used, with standard quality control metrics, to identify CNVs [^7,8^](https://www.zotero.org/google-docs/?L6ACK0).

#### **16p11.2 European Consortium**

MRI data of the EU participants were acquired on two 3T whole-body scanners. 14 carriers of a 16p11.2 deletion and 17 duplication carriers, together with 59 controls (21 familial and 38 unrelated controls) were examined on a Magnetom TIM Trio (Siemens Healthcare, Erlangen, Germany), using a 12-channel RF receive head coil and RF body transmit coil. The remaining 16p11.2 (13 deletions, 6 duplications), 1q11.2 (9 deletions, 7 duplications) carriers and controls (n=38) in the european cohort, were scanned on a Magnetom Prisma Syngo (Siemens Healthcare, Erlangen, Germany) using a 64-channel RF receive head coil and RF body transmit coil. T1-weighted (T1w) anatomical images acquired with the TIM Trio scanner used a Multi-Echo Magnetization Prepared RApid Gradient Echo sequence (ME-MPRAGE: 176 slices; 256×256 matrix; echo time (TE): TE1 = 1.64 ms, TE2 = 3.5 ms, TE3 = 5.36 ms, TE4 = 7.22 ms; repetition time (TR): 2530 ms; flip angle 7°). On the Prisma Syngo scanner, T1w images were acquired using a single-echo MPRAGE sequence (176 slices; 256×256 matrix; TE = 2.39 ms; TR = 2000 ms; flip angle 9°).

#### **Simons Searchlight Consortium**

Data were acquired using multi and single-echo sequences. 176 participants (38 del/ 34 dup 16p11.2 carriers, 2 dup 1q21.1 carriers and 102 familial controls) underwent the research MRI protocol at two imaging core sites on matched 3T Magnetom TIM Trio MRI scanners (Siemens Healthcare, Erlangen, Germany), using the vendor-supplied 32-channel phased-array radio-frequency head coils. 68 participants were scanned at University of California sites (UC) and 108 at the Children Hospital of Philadelphia (CHOP). Structural MRI data included multi-echo T1w ME-MPRAGE using the following parameters: 176 slices, 256×256 matrix, TR = 2530 ms, TI = 1200 ms, TE = 1.64 ms, and flip angle 7°. Clinical MRI images (single-echo) obtained at the phenotyping core sites were also analyzed. The remaining 79 subjects (19 del/ 13 dup 16p11.2 carriers, 12 del/8 dup 1q21.1 carriers and 27 familial controls) were scanned at University of Washington Medical Center, Baylor University Medical Center and Boston Children’s Hospital on two matched 3T Philips Achieva (Philips Healthcare, United States of America) and one unmatched Magnetom TIM Trio scanner (Siemens Healthcare, Erlangen, Germany), respectively. T1w images were acquired using a single-echo MPRAGE sequence and the following parameters: 160 slices; 256×256 matrix; TE = 2.98 ms; TR = 2300 ms; flip angle 9°. All multi-echo images were averaged following a Root-Mean Square (RMS) averaging method.

#### **Brain Canada**

MRI scans for the Brain Canada cohort have been performed at the Montreal Neurological Institute with the same 3T scanner: Magnetom Prisma Syngo (Siemens Healthcare, Erlangen, Germany). Data included 16p11.2 (3 deletions, 3 duplications), 1q11.2 (5 deletions, 1 duplication), 22q11.2 (1 duplication) carriers and controls (n=26) T1w images were acquired using using MPRAGE sequences, scanning protocol description is detailed on this website: http://www.bic.mni.mcgill.ca/users/jlewis/BrainCanada/MCIN/.

#### **UCLA**

Imaging data of 22q11.2 CNV carriers and typically developing (TD) controls were acquired at the University of California, Los Angeles (UCLA). Patients were ascertained from the UCLA or Children's Hospital, Los Angeles Pediatric Genetics, Allergy/Immunology and/or Craniofacial Clinics. We excluded 11 individuals from the analysis due to insufficient quality of the imaging data (cf. Supplementary Methods, quality control). The final 22q11.2 sample includes 144 individuals (71 deletions, 19 duplications and 54 controls). Demographically comparable TD comparison subjects were recruited from the same communities as patients via web-based advertisements and by posting flyers and brochures at local schools, pediatric clinics, and other community sites. Exclusion criteria for all study participants included significant neurological or medical conditions (unrelated to 22q11.2 mutation) that might affect brain structure, history of head injury with loss of consciousness, insufficient fluency in English, and/or substance or alcohol abuse or dependence within the past 6 months. The UCLA Institutional Review Board approved all study procedures and informed consent documents. Scanning was conducted on an identical 3 tesla Siemens Trio MRI scanner with a 12-channel head coil at the University of California at Los Angeles Brain Mapping Center or at the Center for Cognitive Neuroscience.

#### **Cardiff**

Imaging acquisition in Cardiff was performed on a 3 T General Electric HDx MRI system (GE Medical Systems, Milwaukee, WI) using an eight-channel receive-only head RF coil. T1-weighted structural images were acquired with a 3D fast spoiled gradient echo (FSPGR) sequence (TR = 7.8 ms, TE = 3.0 ms, voxel size = 1 mm³ isomorphic). Data included 1 16p11.2 deletion, 1q11.2 (3 deletions, 1 duplication), 22q11.2 (3 deletions, 2 duplications) carriers and 15 controls.

#### **MRI quality control**

All MRI T1w nifti images were visually inspected by the same rater (CM) for head coverage, ghosting and susceptibility artifacts. Images were also screened after segmentation to ensure good tissue classification accuracy. From the clinically ascertained dataset 55 subjects were excluded for insufficient image quality or artifacts while from the non-clinically ascertained dataset 52 subjects were excluded following the same criteria. Quality assurance protocol for Freesurfer based cortical reconstructions led to exclusion of an additional 34 scans. Numbers reported in Table 1 and Supplementary Table 1 are after exclusion.

## List of abbreviations

CNV: Copy Number Variant, ASD: Autism Spectrum Disorders, SCZ: Schizophrenia, VBM: Voxel Based Morphometry, SBM: Surface Based Morphometry, TIV: Total Intracranial Volume, GM: Grey Matter, WM: White Matter, ICV: Intracranial Volume, CT: Cortical Thickness, SA: Surface Area, IQ: Intelligence Quotient, CCA: Canonical Correlation Analysis, PCA: Principal Component Analysis, pLI: Probability of being loss-of-function intolerant.

## Supplementary Method 1: MRI data processing, Voxel-Based Morphometry

We used the probabilistic tissue classification within SPM12 “unified segmentation” framework [^9^](https://www.zotero.org/google-docs/?EzQ1A6) and enhanced tissue priors for optimal delineation of subcortical structures [^10^](https://www.zotero.org/google-docs/?ZT5ODY). The obtained grey and white matter maps were screened for tissue misclassification by the same researcher (CM). Individuals’ gray matter (GM) maps were subsequently spatially registered to the standard Montreal Neurological Institute (MNI) space using DARTELs diffeomorphic registration [^11^](https://www.zotero.org/google-docs/?D83F6p) followed by voxel-based scaling using the Jacobian determinants of the deformation field (i.e., “modulation”). Finally, GM maps were smoothed with a Gaussian of 8mm full-width-at-half-maximum. Parcellation into regions of interest (ROIs) was performed using maximum probability tissue labels (http://www.neuromorphometrics.com/) build using data from the OASIS project (http://www.oasis-brains.org). ROIs’ volumes obtained for each subject were used for PCA and CCA, with the exception of white matter ROIs that were excluded.

## Supplementary Method 2: MRI data processing, Surface-Based Morphometry

ENIGMA quality control procedures (http://enigma.ini.usc.edu/protocols/imaging-protocols/) led to the exclusion of additional 34 scans. Applying FreeSurfer’s recon-all pipeline, estimates of local cortical thickness (CT) and surface area (SA) were calculated at each vertex. Statistical analyses were conducted on each vertex (Freesurfer fsaverage, 327,684 vertices), after spatially smoothing with a Gaussian kernel of 10-mm full width at half maximum (FWHM). In addition, FreeSurfer provided estimates of global brain measures of mean cortical thickness, total surface area per hemisphere, and regional measures based on the Desikan Freesurfer atlas [^12^](https://www.zotero.org/google-docs/?QYTI0T).

## Supplementary Method 3: Statistical analysis for global brain measures

Within the two cohort ascertainments, we used ANOVA design to compare group means of global brain measures. P-value correction for multiple comparisons was performed with the Tukey honest significant differences test. Wilcoxon rank sum and signed rank tests were used to compare distributions between deletions and duplications of each CNV.

## Supplementary Method 4: Voxel-based measures and statistical analyses

GM maps were entered as dependent variables, group - as independent variable (variance between groups was set as unequal) and age, age^2^, sex, scanning site, total grey matter volume - as fixed effects. After model estimation, post hoc contrasts were calculated to compare each CNV group to the corresponding control group. VBM results on grey matter are significant at p<0.05, FWE corrected [^2–4^](https://www.zotero.org/google-docs/?FbGZdl). We tested for significant effects in a conjunction analysis across the pre-defined contrasts.

## Supplementary Method 5: Surface-based measures and statistical analyses

In parallel to VBM, we used surface-based GLM-based analysis to test differences in CT and SA (SurfStat toolbox [^13^](https://www.zotero.org/google-docs/?aXSX7u)). Each GLM used the surface feature as the dependent variable, the groups - as independent variable, which were adjusted for age, age^2^, sex, site, and Total-SA/Mean-CT. Post-hoc contrasts compared each CNV group against controls, including estimation of Cohen’s d effect size estimates from t-values [^14^](https://www.zotero.org/google-docs/?op9u06). False Discovery Rate (FDR), with p-value at 0.05 was applied to control for false positive errors due to multiple comparisons.

## Supplementary Method 6: Spin permutation testing

The spin permutation test provides a null hypothesis quantifying the probability of observing by chance a dice index or correlation value, while controlling for spatial auto-correlations inherent in neuroimaging data. This method has been established previously in [^15,16^](https://www.zotero.org/google-docs/?gWuJl5). To perform the spin permutation test, we applied 10,000 random surface-based rotations of XYZ to a given map, generating random maps while preserving the relative spatial organization of the vertices. We then generate a null distribution of dice indices or correlation values for deletion and duplication convergence patterns or deletion-duplication anti-correlation respectively. An empirical p-value (p-value_SPIN_) was then obtained as the proportion of 10,000 null dice-indices or correlations that had a higher value than the observed value. P-value_SPIN_ < 0.05 was used as a statistical significance threshold [^15,16^](https://www.zotero.org/google-docs/?rITdfv).

## Supplementary Method 7: Label shuffling

We additionally tested the overlap significance by performing permutation of control and CNV labels, generating empirical null distributions and calculating dice index distribution with regard to the convergence pattern. We performed permutation for control and CNV labels (7 groups, clinically ascertained) 10,000 times, and calculated the same overlap using the 15^th^ and 85^th^ percentile of Cohen’s d values (the top 15% of positive and negative effect sizes) between the three random groups. For 15q11.2 CNVs (non-clinically ascertained), we shuffled control and CNV labels 10,000 times. We then calculated the same overlap using the 15^th^ and 85^th^ percentile of Cohen’s d values (the top 15% of positive and negative effect sizes) with 3-deletion convergence pattern (cf. above). To generate empirical null distributions to obtain p-values (p-value_SHUFFLE_) for significance testing, we calculated dice index distribution w.r.t the convergence pattern. Similarly, for a new map, we calculated the dice index with respect to the convergence pattern and 10,000 random patterns generated above. An empirical p-value (p-value_SHUFFLE_) was then obtained based on the proportion of 10,000 random map based dice-indices that had a higher value than the observed value. p-value_SHUFFLE_ < 0.05 was used as a statistical significance threshold [^15,16^](https://www.zotero.org/google-docs/?ZEXKG4).

## Supplementary Method 8: Multi-view pattern-learning analysis

Canonical correlation analysis (CCA) allows a single integrated multivariate analysis to simultaneously co-analyze brain morphometry measures along with the Genomic loci information (CNV status). This aims to identify symmetric linear relations between the two sets of variables. In our study, the first variable set (X) represents the CNV status of a subject, based on encoding with -1 (deletion) 0 (normal diploid) or +1 (duplication). All regional volume measures were normalized by de-meaning to 0 and re-scaling to 1. That is, the set of CNV carrier status was jointly encoded, capturing genomic loci. The second variable set (Y) was encoded as a matrix of 130 regional grey matter volumes (68 Desikan ROIs for SA, and CT) adjusted for total grey matter volume (Total SA, and Mean CT for SA, and CT). The brain volume measures in variable set Y were fed into CCA after a confound-removal procedure including age, age^2^, sex, site, and total grey matter analogous to previous studies [^17–19^](https://www.zotero.org/google-docs/?Q9vTeI).

Our CCA strategy involved finding pairs of canonical vectors u and v that maximize the correlation in the embedding space (canonical variates) between a linear combination of the set of CNV indicators (X) and a linear combination of regional volumes (Y). In other words, canonical vectors project high dimensional variable sets onto a new low-rank space defined by the linear combination of original variables, called canonical variates. The Canonical Correlation, calculated as Pearson’s correlation (r) between X and Y canonical variates, can be seen as a metric of joint information reduction and serves as a performance measure for CCA. In our study, CCA identifies modes of coherent co-variation that jointly characterize how CNVs and patterns of regional volumes systematically co-occur across subjects. We refer to these modes of co-variation as ‘CCA dimensions’ or ‘gene-morphology dimensions’.

The statistical significance of the derived dominant modes of gene-morphology co-variation was determined by null hypothesis testing based on a non-parametric permutation procedure [^17^](https://www.zotero.org/google-docs/?jLaz8l). Relying on minimal modeling assumptions [^20^](https://www.zotero.org/google-docs/?1iWUpt), an empirical null distribution representing the absence of correlation between CNVs and brain morphometry. 1,000 gene-volume correlations were computed after permuting the original data such that the CNV status and set of region volume expressions were unrelated across individuals. In 1,000 permutation iterations, the CNV labels were held constant, while the regional volumes were subject to participant-wise random shuffling. The CCA analysis was rerun for each permutation iteration (the maximum possible correlation coefficient). An original CCA dimension was declared statistically significant if the associated canonical correlation values exceeded the 99% percentile of the null distribution (i.e., p<0.05).

##
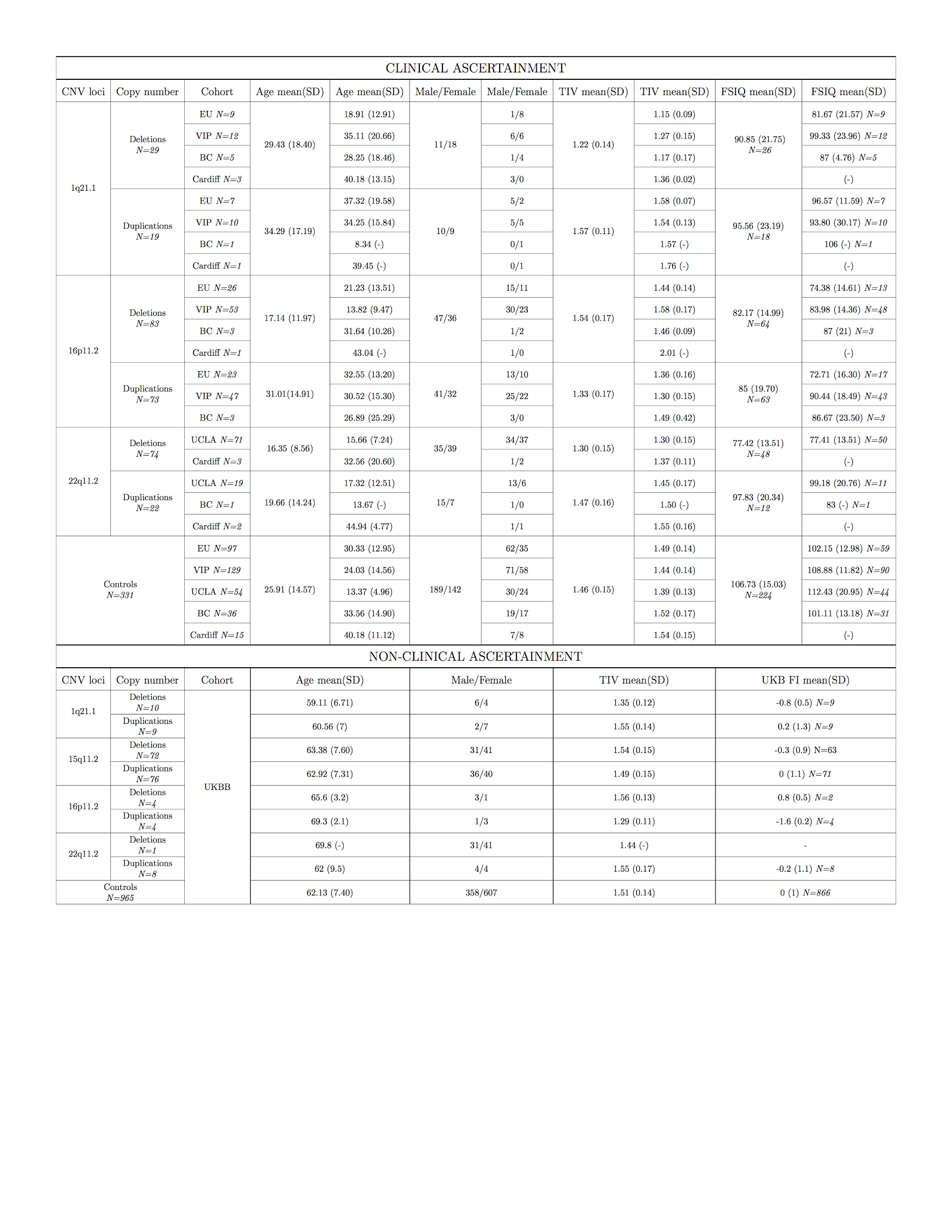
Supplementary Table 1 : Demographics

Legend: EU: 16p11.2 European Consortium, VIP: Simons Searchlight Consortium, BC: Brain Canada, CNV: Copy Number Variant, SD: Standard deviation, TIV: total intracranial volume, FSIQ: Full-scale IQ, UKB FI: UK Biobank fluid intelligence, ASD: Autism Spectrum Disorders, SCZ: schizophrenia (including * ICD10 code F25.9 Schizoaffective disorder, unspecified). CNV carriers and controls from the clinically ascertained group come from 5 different cohorts, while non-clinically ascertained participants were identified in the UK Biobank. UK Biobank fluid intelligence scores (UKB field:20016) were adjusted for age, age^2^, sex, site, and then z-scored. 16p11.2 and 22q11.2 from the UKBB were not included in the VBM and SBM due to small sample size. Data for 16p11.2 and 22q11.2 deletions and duplications from the VIP, EU and UCLA datasets were previously published in Martin-Brevet *et al.* 2018 and Lin *et al.* 2017 [^1,4^](https://www.zotero.org/google-docs/?5SR6jd).

|  | | total Surface Area | | mean Cortical Thickness | |
| --- | --- | --- | --- | --- | --- |
|  |  | Z-score mean | Z-score sd | Z-score mean | Z-score sd |
| clinical ascertainment | 1q21.1 deletion | -1.96 | 0.65 | 0.37 | 0.94 |
|  | 1q21.1 duplication | 1.16 | 1.01 | 0.13 | 1.76 |
|  | 16p11.2 deletion | 0.52 | 1.24 | -0.26 | 1.07 |
|  | 16p11.2 duplication | -1.17 | 1.00 | -0.51 | 0.89 |
|  | 22q11.2 deletion | -0.97 | 0.98 | 0.41 | 1.16 |
|  | 22q11.2 duplication | 0.44 | 0.94 | -0.81 | 0.99 |
|  | controls | 0.00 | 1.00 | 0.00 | 1.00 |
| non-clinical ascertainment | 1q21.1 deletion | -2.06 | 0.56 | 0.58 | 0.89 |
|  | 1q21.1 duplication | 0.83 | 0.99 | 0.10 | 1.18 |
|  | 15q11.2 deletion | -0.13 | 0.99 | 0.38 | 0.97 |
|  | 15q11.2 duplication | -0.17 | 1.09 | -0.29 | 0.92 |
|  | 16p11.2 deletion | -0.70 | 0.24 | 0.87 | 1.18 |
|  | 16p11.2 duplication | 1.24 | 1.04 | -1.47 | 0.97 |
|  | 22q11.2 deletion | -0.58 | NA | 0.86 | NA |
|  | 22q11.2 duplication | 0.04 | 0.63 | 0.04 | 1.10 |
|  | controls | 0.00 | 1.00 | 0.00 | 1.00 |
|  | | Total Brain Volume | | total Grey Matter | |
|  |  | Z-score mean | Z-score sd | Z-score mean | Z-score sd |
| clinical ascertainment | 1q21.1 deletion | -1.72 | 0.82 | -1.85 | 0.75 |
|  | 1q21.1 duplication | 0.89 | 0.83 | 1.02 | 1.06 |
|  | 16p11.2 deletion | 0.61 | 1.23 | 0.40 | 1.24 |
|  | 16p11.2 duplication | -1.10 | 1.12 | -1.12 | 1.09 |
|  | 22q11.2 deletion | -0.60 | 0.95 | -0.75 | 0.98 |
|  | 22q11.2 duplication | 0.36 | 1.15 | 0.40 | 1.08 |
|  | controls | 0.00 | 1.00 | 0.00 | 1.00 |
| non-clinical ascertainment | 1q21.1 deletion | -1.91 | 0.97 | -2.03 | 0.56 |
|  | 1q21.1 duplication | 0.67 | 1.28 | 0.70 | 1.31 |
|  | 15q11.2 deletion | -1.64 | 0.93 | 0.09 | 1.08 |
|  | 15q11.2 duplication | 0.17 | 1.22 | -0.35 | 1.12 |
|  | 16p11.2 deletion | -0.11 | 0.71 | -0.17 | 0.83 |
|  | 16p11.2 duplication | -1.78 | 0.59 | -1.31 | 0.59 |
|  | 22q11.2 deletion | 0.17 | NA | -0.07 | NA |
|  | 22q11.2 duplication | -0.32 | 0.88 | 0.35 | 0.69 |
|  | controls | 0.00 | 1.00 | 0.00 | 1.00 |

## Supplementary Table 2: Mean and standard deviation for each CNV on global metrics.

Legend: Mean and standard deviations of global metrics z-scores for each CNV group. These data are represented in Figure 1

|  | Median Cohen’s d  5th percentile | Median Cohen’s d 95th percentile | % of voxels surviving FWE | % of voxels with negative effects surviving FWE | % of voxels with positive effects surviving FWE |
| --- | --- | --- | --- | --- | --- |
| 1q21.1 deletion | -0.29 | 0.30 | 1.49% | 0.64% | 0.84% |
| 1q21.1 duplication | -0.23 | 0.22 | 0.04% | 0.01% | 0.03% |
| 16p11.2 deletion | -0.48 | 0.65 | 17.13% | 8.03% | 9.09% |
| 16p11.2 duplication | -0.37 | 0.25 | 2.84% | 2.66% | 0.17% |
| 22q11.2 deletion | -0.45 | 0.59 | 14.81% | 7.28% | 7.52% |
| 22q11.2 duplication | -0.23 | 0.27 | 0.32% | 0.06% | 0.26% |
| 1q21.1 deletion UKBB | -0.14 | 0.17 | 0.31% | - | 0.31% |
| 1q21.1 duplication UKBB | -0.14 | 0.15 | 0.04% | - | 0.04% |
| 15q11.2 deletion UKBB | -0.13 | 0.14 | 0.02% | 0.02% | - |
| 15q11.2 duplication UKBB | -0.12 | 0.14 | 0.002% | - | 0.002% |

## Supplementary Table 3: CNV effect sizes on local grey matter volume changes

Legend: First and second column report medians of 5th and 95th percentiles of Cohen’s d distributions. Columns three to five report voxels surviving FWE error correction.

| Desikan-Killiany Atlas | | Tail1 (<15%) | | | Tail2 (>85%) | | |
| --- | --- | --- | --- | --- | --- | --- | --- |
| ROIname | #vertices | SA-CT | SA-VBMfs | CT-VBMfs | SA-CT | SA-VBMfs | CT-VBMfs |
| bankssts | 4333 | 0 | 0 | 0 | 0 | 0 | 0 |
| caudalanteriorcingulate | 3047 | 7 | 61 | 28 | 0 | 0 | 0 |
| caudalmiddlefrontal | 7230 | 0 | 0 | 0 | 0 | 0 | 0 |
| cuneus | 3268 | 0 | 0 | 0 | 0 | 0 | 0 |
| entorhinal | 2004 | 0 | 0 | 0 | 0 | 0 | 0 |
| fusiform | 9375 | 0 | 0 | 0 | 0 | 0 | 0 |
| inferiorparietal | 17547 | 0 | 0 | 0 | 0 | 0 | 0 |
| inferiortemporal | 8613 | 0 | 0 | 0 | 0 | 0 | 0 |
| isthmuscingulate | 4919 | 0 | 0 | 0 | 0 | 52 | 0 |
| lateraloccipital | 12342 | 0 | 0 | 0 | 0 | 0 | 0 |
| lateralorbitofrontal | 8542 | 0 | 0 | 0 | 0 | 69 | 0 |
| lingual | 8099 | 0 | 0 | 0 | 0 | 0 | 0 |
| medialorbitofrontal | 5454 | 0 | 0 | 0 | 0 | 0 | 0 |
| middletemporal | 9509 | 0 | 0 | 0 | 0 | 0 | 0 |
| parahippocampal | 3580 | 0 | 0 | 91 | 0 | 0 | 0 |
| paracentral | 7125 | 0 | 0 | 0 | 0 | 0 | 0 |
| parsopercularis | 5591 | 0 | 0 | 0 | 0 | 5 | 0 |
| parsorbitalis | 1902 | 0 | 0 | 0 | 0 | 0 | 0 |
| parstriangularis | 4426 | 0 | 0 | 0 | 0 | 0 | 0 |
| pericalcarine | 3735 | 0 | 0 | 0 | 0 | 0 | 0 |
| postcentral | 18657 | 0 | 0 | 0 | 0 | 0 | 0 |
| posteriorcingulate | 6260 | 0 | 0 | 0 | 0 | 0 | 0 |
| precentral | 21445 | 0 | 0 | 0 | 0 | 0 | 0 |
| precuneus | 15283 | 0 | 0 | 0 | 0 | 14 | 0 |
| rostralanteriorcingulate | 2401 | 0 | 0 | 0 | 0 | 0 | 0 |
| rostralmiddlefrontal | 15107 | 0 | 0 | 0 | 0 | 0 | 0 |
| superiorfrontal | 24057 | 0 | 0 | 0 | 0 | 0 | 0 |
| superiorparietal | 20678 | 0 | 0 | 0 | 0 | 0 | 0 |
| superiortemporal | 14139 | 0 | 0 | 0 | 0 | 0 | 0 |
| supramarginal | 16750 | 0 | 0 | 0 | 0 | 0 | 0 |
| frontalpole | 641 | 0 | 0 | 0 | 0 | 0 | 0 |
| temporalpole | 1656 | 0 | 0 | 0 | 0 | 0 | 0 |
| transversetemporal | 1845 | 0 | 0 | 0 | 0 | 0 | 0 |
| insula | 10319 | 0 | 0 | 0 | 0 | 123 | 0 |

## Supplementary Table 4: Intersection of overlap maps across SA-CT-VBM

Legend: Table showing the number of vertices (sum of left and right hemispheres) intersecting between pairs of SA-CT-VBM overlap maps (Deletions of 1q21.1, 15q11.2, 16p11.2, and 22q11.2) for both tails <15% (left side) and >85% (right side), mapped to Desikan Regions of Interests (ROIs). See Supplementary Figure 16 for brain maps. VBM overlap maps are projected onto fsaverage using mri_vol2surf function in Freesurfer, and are restricted to cortex for comparison. Cells are highlighted for ease of visualization. Only 7 vertices intersect across all three - SA-CT-VBMfs overlap maps - for tail 1 (<15%). No intersection between pairs of SA-CT-VBM overlap maps is observed for duplications and hence not included. SA: Surface Area; CT: Cortical Thickness; VBM: Voxel Based Morphometry; #vertices: number of vertices for a ROI in fsaverage (left and right hemisphere sum in the above table).

|  | Median Cohen’s d  5th percentile | Median Cohen’s d 95th percentile | % of vertices surviving FDR (q<0.05) | % of vertices with negative effects surviving FDR (q<0.05) | % of vertices with positive effects surviving FDR (q<0.05) |
| --- | --- | --- | --- | --- | --- |
| 1q21.1 deletion | -0.21 | 0.21 | 0.31% | - | 0.31% |
| 1q21.1 duplication | -0.18 | 0.20 | 0.06% | - | 0.06% |
| 16p11.2 deletion | -0.51 | 0.63 | 42.45% | 19.97% | 22.48% |
| 16p11.2 duplication | -0.35 | 0.30 | 13.91% | 8.81% | 5.10% |
| 22q11.2 deletion | -0.44 | 0.49 | 34.07% | 14.60% | 19.47% |
| 22q11.2 duplication | -0.18 | 0.21 | 1.31% | 0.31% | 1.00% |
| 1q21.1 deletion UKBB | -0.11 | 0.11 | - | - | - |
| 1q21.1 duplication UKBB | -0.12 | 0.15 | 0.10% | 0.03% | 0.07% |
| 15q11.2 deletion UKBB | -0.12 | 0.13 | 0.07% | - | 0.07% |
| 15q11.2 duplication UKBB | -0.13 | 0.14 | - | - | - |

## Supplementary Table 5: CNV effect sizes on local Surface Area changes

Legend: First and second column report medians of 5th and 95th percentiles of Cohen’s d distributions. Columns three to five report vertices surviving FDR correction (q<0.05).

|  | Median Cohen’s d  5th percentile | Median Cohen’s d 95th percentile | % of vertices surviving FDR (q<0.05) | % of vertices with negative effects surviving FDR (q<0.05) | % of vertices with positive effects surviving FDR (q<0.05) |
| --- | --- | --- | --- | --- | --- |
| 1q21.1 deletion | -0.21 | 0.24 | 2.44% | 0.55 | 1.89% |
| 1q21.1 duplication | -0.20 | 0.18 | - | - | - |
| 16p11.2 deletion | -0.31 | 0.35 | 18.31% | 8.67% | 9.64% |
| 16p11.2 duplication | -0.27 | 0.26 | 6.88% | 4.02% | 2.86% |
| 22q11.2 deletion | -0.44 | 0.39 | 27.96% | 18.52% | 9.44% |
| 22q11.2 duplication | -0.22 | 0.20 | 0.71% | 0.46% | 0.25% |
| 1q21.1 deletion UKBB | -0.12 | 0.14 | - | - | - |
| 1q21.1 duplication UKBB | -0.23 | 0.08 | 19.23% | 19.23% | - |
| 15q11.2 deletion UKBB | -0.12 | 0.14 | - | - | - |
| 15q11.2 duplication UKBB | -0.11 | 0.12 | - | - | - |

## Supplementary Table 6: CNV effect sizes on local Cortical Thickness changes

Legend: First and second column report medians of 5th and 95th percentiles of Cohen’s d distributions. Columns three to five report vertices surviving FDR correction (q<0.05).

|  | Ratio del/dup (GM) | F test del/dup (GM) | Degrees of freedom df1-df2 (GM) | p-value *F* test (GM) | Ratio del/dup (SA) | F test del/dup (SA) | Degrees of freedom df1-df2 (SA) | p-value *F* test (SA) |
| --- | --- | --- | --- | --- | --- | --- | --- | --- |
| 1q21.1 | 1.297 | 1.682 | 416407-416408 | 0 | 1.087 | 1.174 | 327683-327683 | 0 |
| 16p11.2 | 1.846 | 3.485 | 416408-416408 | 0 | 1.772 | 3.141 | 327683-327683 | 0 |
| 22q11.2 | 2.019 | 4.0748 | 416408-416407 | 0 | 2.267 | 5.139 | 327683-327683 | 0 |
| ukb 1q21.1 | 1.013 | 1.025 | 385045-385046 | 0 | 0.795 | 0.631 | 327683-327683 | 0 |
| ubk 15q11.2 | 1.071 | 1.148 | 385045-385045 | 0 | 0.944 | 0.892 | 327683-327683 | 0 |

## Supplementary Table 7: Within loci comparison of Cohen’s d variances between deletions and duplications

Legend: Standard deviation ratio and two-sample *F* test for equal variances on Cohen’s D distributions of GM volume and SA. Second and sixth columns report standard deviations ratios between deletion and duplication Cohen’s d distributions. Column 3-5 and 7-9 report two-sample F test statistics for GM volume and SA for each loci.

|  | 1q21.1 | 16p11.2 | 22q11.2 | ukb1q21.1 | ukb15q11.2 |
| --- | --- | --- | --- | --- | --- |
| 1q21.1 | -0.187 | -0.058 | 0.258* | 0.213* | 0.150 |
| 16p11.2 | 0.141 | -0.626* | -0.141 | 0.035 | -0.025 |
| 22q11.2 | 0.035 | 0.208 | -0.232* | -0.006 | -0.061 |
| ukb1q21.1 | 0.535* | 0.064 | 0.058 | -0.384* | 0.083 |
| ukb15q11.2 | -0.138 | 0.020 | 0.173 | -0.175 | -0.184 |

## Supplementary Table 8: Brain-wide Cohen’s d map Correlations for VBM

Legend: Pearson correlation between Cohen’s d maps (brain wide) for Deletion-Duplication (uiagonal, purple cells), Deletion-Deletion (lower triangle, red cells), and Duplication-Duplication (upper triangle, blue cells) for clinical and non-clinical CNVs. ukb15q11.2 and ukb1q21.1 correspond to UKBB CNV carriers. * Bonferoni correction, p-value_SPIN_ <0.002 (=0.05/25).

|  | 1q21.1 | 16p11.2 | 22q11.2 | ukb1q21.1 | ukb15q11.2 |
| --- | --- | --- | --- | --- | --- |
| 1q21.1 | -0.147 | 0.232* | -0.030 | 0.318* | 0.021 |
| 16p11.2 | 0.136 | -0.551* | -0.006 | 0.083 | 0.025 |
| 22q11.2 | 0.111 | 0.133 | -0.139 | 0.035 | 0.050 |
| ukb1q21.1 | 0.332* | 0.118 | -0.143 | -0.275* | 0.041 |
| ukb15q11.2 | 0.058 | 0.054 | 0.178 | -0.035 | -0.231* |

## Supplementary Table 9: Cortex-wide Cohen’s d map Correlations for SA

Legend: Pearson correlation between Cohen’s d maps (coretx wide) for Deletion-Duplication (diagonal, purple cells), Deletion-Deletion (lower triangle, red cells), and Duplication-Duplication (upper triangle, blue cells) for clinical and non-clinical CNVs. ukb15q11.2 and ukb1q21.1 correspond to UKBB CNV carriers. * Bonferoni correction, p-value_SPIN_ <0.002 (=0.05/25).

|  | 1q21.1 | 16p11.2 | 22q11.2 | ukb1q21.1 | ukb15q11.2 |
| --- | --- | --- | --- | --- | --- |
| 1q21.1 | -0.074 | 0.083 | 0.165 | 0.059 | 0.041 |
| 16p11.2 | 0.132 | -0.169 | -0.094 | -0.068 | 0.066 |
| 22q11.2 | -0.037 | 0.032 | 0.012 | 0.198 | -0.044 |
| ukb1q21.1 | 0.381* | 0.123 | -0.130 | -0.103 | -0.009 |
| ukb15q11.2 | 0.032 | 0.074 | -0.016 | 0.115 | -0.092 |

## Supplementary Table 10: Cortex-wide Cohen’s d map Correlations for CT

Legend: Pearson correlation between Cohen’s d maps (coretx wide) for Deletion-Duplication (diagonal, purple cells), Deletion-Deletion (lower triangle, red cells), and Duplication-Duplication (upper triangle, blue cells) for clinical and non-clinical CNVs. ukb15q11.2 and ukb1q21.1 correspond to UKBB CNV carriers. * Bonferoni correction, p-value_SPIN_ <0.002 (=0.05/25).

| Neuromorphometric brain regions | 1q21.1 del | 1q21.1 dup | 16p11.2 del | 16p11.2 dup | 22q11.2 del | 22q11.2 dup | 15q11.2 del | 15q11.2 dup |
| --- | --- | --- | --- | --- | --- | --- | --- | --- |
| X3rd.Ventricle | 0.01 | 0.06 | 0.14 | 0.05 | 0.26 | 0.12 | 0.35 | -0.17 |
| X4th.Ventricle | 0.47 | -0.58 | -0.07 | 0.00 | 0.02 | -0.06 | 0.10 | -0.10 |
| R.Accumbens.Area | 0.15 | 0.53 | -0.50 | 0.14 | 0.97 | -0.87 | -0.24 | 0.08 |
| L.Accumbens.Area | 0.01 | 0.27 | -0.39 | 0.09 | 0.95 | -0.99 | -0.11 | 0.07 |
| R.Amygdala | 0.26 | 0.20 | 0.13 | -0.03 | 0.16 | 0.28 | -0.23 | 0.09 |
| L.Amygdala | 0.40 | -0.19 | 0.04 | -0.10 | 0.11 | 0.09 | -0.28 | 0.13 |
| Brain.Stem | 0.12 | -0.32 | -0.02 | -0.18 | -0.46 | 0.68 | -0.27 | 0.20 |
| R.Caudate | 0.27 | -0.12 | -0.20 | -0.22 | 0.63 | -0.60 | 0.14 | -0.09 |
| L.Caudate | 0.29 | -0.32 | -0.14 | -0.35 | 0.55 | -0.65 | 0.01 | -0.06 |
| R.Cerebellum.Exterior | 0.02 | -0.34 | -0.48 | 0.17 | -0.47 | 0.73 | -0.20 | -0.15 |
| L.Cerebellum.Exterior | 0.08 | -0.34 | -0.46 | 0.11 | -0.50 | 0.85 | -0.20 | -0.18 |
| CSF | -0.14 | 0.15 | 0.08 | 0.09 | 0.48 | -0.14 | 0.34 | -0.23 |
| R.Hippocampus | 0.56 | -0.48 | -0.08 | 0.02 | 0.01 | 0.33 | -0.36 | -0.04 |
| L.Hippocampus | 0.58 | -0.65 | -0.15 | 0.13 | 0.08 | -0.04 | -0.36 | -0.01 |
| R.Inf.Lat.Vent | -0.22 | -0.06 | 0.12 | 0.18 | 0.34 | -0.22 | 0.06 | -0.14 |
| L.Inf.Lat.Vent | -0.09 | -0.01 | 0.16 | 0.19 | 0.31 | -0.24 | 0.02 | -0.12 |
| R.Lateral.Ventricle | -0.07 | 0.49 | -0.05 | 0.43 | 0.44 | -0.03 | 0.28 | -0.17 |
| L.Lateral.Ventricle | -0.04 | 0.52 | -0.03 | 0.40 | 0.38 | 0.05 | 0.28 | -0.21 |
| R.Pallidum | 0.18 | -0.61 | -0.05 | -0.31 | 0.12 | -0.19 | -0.17 | 0.17 |
| L.Pallidum | 0.03 | -0.70 | -0.05 | -0.39 | 0.13 | -0.29 | -0.16 | 0.14 |
| R.Putamen | 0.45 | 0.01 | -0.10 | -0.29 | 0.02 | -0.24 | -0.24 | -0.02 |
| L.Putamen | 0.42 | 0.02 | -0.16 | -0.28 | 0.05 | -0.22 | -0.33 | 0.00 |
| R.Thalamus.Proper | 0.64 | -0.44 | 0.17 | -0.56 | 0.18 | -0.01 | 0.01 | 0.19 |
| L.Thalamus.Proper | 0.60 | -0.54 | 0.20 | -0.62 | 0.22 | -0.10 | -0.06 | 0.21 |
| R.Ventral.DC | 0.42 | -0.30 | 0.27 | -0.24 | 0.00 | 0.40 | -0.31 | 0.33 |
| L.Ventral.DC | 0.38 | -0.41 | 0.26 | -0.33 | 0.01 | 0.32 | -0.29 | 0.31 |
| Optic.Chiasm | -0.06 | 0.24 | -0.52 | 0.20 | -0.03 | 0.45 | 0.00 | -0.23 |
| Cerebellar.Vermal.Lobules.I.V | 0.02 | -0.44 | -0.19 | -0.17 | -0.06 | 0.39 | -0.01 | -0.27 |
| Cerebellar.Vermal.Lobules.VI.VII | -0.01 | -0.25 | -0.37 | -0.13 | -0.31 | 0.44 | -0.14 | -0.12 |
| Cerebellar.Vermal.Lobules.VIII.X | 0.44 | -0.43 | 0.00 | -0.17 | -0.39 | 0.76 | -0.13 | -0.03 |
| L.Basal.Forebrain | 0.09 | 0.70 | 0.30 | -0.04 | 0.55 | -0.47 | -0.12 | 0.15 |
| R.Basal.Forebrain | 0.13 | 0.65 | 0.29 | 0.08 | 0.61 | -0.38 | -0.14 | 0.29 |
| R.ACgG.anterior.cingulate.gyrus | -0.49 | 0.41 | -0.15 | -0.20 | -0.22 | 0.40 | -0.10 | 0.05 |
| L.ACgG.anterior.cingulate.gyrus | -0.85 | 0.72 | -0.20 | 0.03 | -0.15 | 0.41 | -0.02 | 0.07 |
| R.AIns.anterior.insula | -0.21 | 0.16 | 0.75 | -0.50 | 0.65 | -0.57 | 0.50 | -0.14 |
| L.AIns.anterior.insula | -0.15 | 0.08 | 0.66 | -0.51 | 0.72 | -0.60 | 0.35 | -0.07 |
| R.AOrG.anterior.orbital.gyrus | -0.35 | 0.48 | -0.09 | -0.14 | 0.07 | -0.09 | -0.08 | 0.18 |
| L.AOrG.anterior.orbital.gyrus | -0.50 | 0.06 | 0.01 | -0.06 | 0.07 | 0.09 | 0.03 | 0.18 |
| R.AnG.angular.gyrus | -0.11 | 0.36 | -0.48 | 0.25 | -0.29 | 0.17 | -0.06 | -0.12 |
| L.AnG.angular.gyrus | 0.11 | -0.06 | -0.65 | 0.32 | -0.28 | 0.26 | -0.10 | -0.18 |
| R.Calc.calcarine.cortex | 0.02 | 0.21 | 0.85 | -0.59 | -0.46 | 0.58 | -0.22 | -0.06 |
| L.Calc.calcarine.cortex | 0.08 | 0.11 | 0.83 | -0.56 | -0.50 | 0.46 | -0.28 | -0.01 |
| R.CO.central.operculum | -0.30 | 0.25 | 0.03 | 0.02 | 0.29 | -0.25 | 0.13 | 0.06 |
| L.CO.central.operculum | -0.27 | 0.18 | 0.31 | -0.16 | 0.45 | -0.15 | 0.17 | 0.06 |
| R.Cun.cuneus | 0.06 | 0.14 | 0.20 | -0.19 | -0.59 | 0.50 | -0.20 | -0.20 |
| L.Cun.cuneus | -0.06 | 0.22 | 0.29 | -0.43 | -0.69 | 0.74 | -0.19 | -0.24 |
| R.Ent.entorhinal.area | 0.17 | 0.47 | 0.08 | 0.11 | -0.06 | 0.50 | -0.11 | 0.07 |
| L.Ent.entorhinal.area | 0.17 | 0.31 | -0.03 | 0.06 | -0.16 | 0.56 | -0.23 | -0.02 |
| R.FO.frontal.operculum | -0.10 | -0.11 | 0.40 | -0.42 | 0.26 | -0.18 | 0.05 | -0.10 |
| L.FO.frontal.operculum | -0.04 | -0.24 | 0.36 | -0.45 | 0.24 | -0.26 | -0.07 | 0.02 |
| R.FRP.frontal.pole | -0.41 | 0.80 | -0.45 | 0.07 | 0.27 | -0.07 | 0.21 | -0.01 |
| L.FRP.frontal.pole | -0.40 | 0.52 | -0.27 | 0.12 | 0.29 | -0.11 | 0.24 | -0.03 |
| R.FuG.fusiform.gyrus | -0.26 | 0.24 | -0.41 | 0.40 | -0.28 | 0.66 | -0.03 | -0.22 |
| L.FuG.fusiform.gyrus | -0.22 | 0.23 | -0.47 | 0.30 | -0.40 | 0.65 | -0.16 | -0.23 |
| R.GRe.gyrus.rectus | -0.64 | 0.70 | -0.51 | -0.04 | 0.25 | -0.47 | 0.11 | 0.08 |
| L.GRe.gyrus.rectus | -0.61 | 0.49 | -0.27 | -0.10 | 0.40 | -0.50 | 0.00 | 0.00 |
| R.IOG.inferior.occipital.gyrus | -0.27 | 0.28 | 0.31 | -0.09 | -0.35 | 0.59 | 0.05 | -0.10 |
| L.IOG.inferior.occipital.gyrus | -0.04 | 0.49 | 0.06 | -0.20 | -0.10 | 0.30 | 0.09 | -0.27 |
| R.ITG.inferior.temporal.gyrus | -0.38 | -0.17 | -0.28 | 0.43 | -0.31 | 0.63 | 0.04 | -0.22 |
| L.ITG.inferior.temporal.gyrus | -0.36 | -0.08 | -0.25 | 0.36 | -0.32 | 0.63 | -0.04 | -0.37 |
| R.LiG.lingual.gyrus | 0.35 | 0.04 | 0.45 | -0.43 | -0.43 | 0.76 | -0.09 | -0.31 |
| L.LiG.lingual.gyrus | 0.25 | -0.04 | 0.29 | -0.47 | -0.50 | 0.64 | -0.14 | -0.26 |
| R.LOrG.lateral.orbital.gyrus | -0.37 | 0.10 | 0.14 | -0.26 | 0.07 | 0.07 | -0.07 | 0.07 |
| L.LOrG.lateral.orbital.gyrus | -0.49 | -0.27 | 0.17 | -0.13 | 0.06 | 0.14 | -0.14 | 0.25 |
| R.MCgG.middle.cingulate.gyrus | -0.55 | 0.79 | -0.74 | 0.11 | -0.55 | 0.80 | -0.35 | 0.05 |
| L.MCgG.middle.cingulate.gyrus | -0.91 | 0.88 | -0.81 | 0.21 | -0.62 | 0.77 | -0.34 | 0.00 |
| R.MFC.medial.frontal.cortex | -0.70 | 0.69 | -0.16 | 0.11 | 0.21 | 0.11 | 0.18 | 0.14 |
| L.MFC.medial.frontal.cortex | -0.69 | 0.76 | -0.03 | 0.08 | 0.27 | 0.17 | 0.20 | 0.12 |
| R.MFG.middle.frontal.gyrus | -0.90 | 0.64 | -0.55 | 0.32 | 0.14 | -0.17 | -0.05 | 0.06 |
| L.MFG.middle.frontal.gyrus | -1.00 | 0.68 | -0.53 | 0.32 | 0.21 | -0.35 | -0.09 | 0.00 |
| R.MOG.middle.occipital.gyrus | -0.04 | 0.14 | -0.03 | -0.04 | -0.22 | 0.43 | 0.07 | -0.05 |
| L.MOG.middle.occipital.gyrus | 0.14 | 0.37 | -0.36 | -0.05 | -0.15 | 0.20 | -0.11 | -0.13 |
| R.MOrG.medial.orbital.gyrus | -0.36 | 0.52 | 0.09 | -0.37 | 0.21 | -0.20 | -0.01 | 0.13 |
| L.MOrG.medial.orbital.gyrus | -0.26 | 0.37 | 0.13 | -0.35 | 0.25 | -0.40 | 0.02 | 0.11 |
| R.MPoG.postcentral.gyrus.medial.segment | 0.13 | 0.18 | -0.23 | -0.04 | -0.24 | 0.21 | -0.29 | 0.03 |
| L.MPoG.postcentral.gyrus.medial.segment | -0.06 | 0.41 | -0.20 | 0.12 | -0.18 | 0.21 | -0.10 | 0.08 |
| R.MPrG.precentral.gyrus.medial.segment | -0.37 | 0.36 | -0.40 | 0.14 | -0.41 | 0.30 | -0.24 | 0.07 |
| L.MPrG.precentral.gyrus.medial.segment | -0.35 | 0.61 | -0.45 | 0.05 | -0.48 | 0.47 | -0.18 | 0.04 |
| R.MSFG.superior.frontal.gyrus.medial.segment | -0.75 | 0.78 | -0.38 | 0.27 | -0.03 | -0.02 | 0.11 | 0.11 |
| L.MSFG.superior.frontal.gyrus.medial.segment | -0.72 | 0.69 | -0.37 | 0.15 | -0.02 | -0.01 | 0.10 | 0.09 |
| R.MTG.middle.temporal.gyrus | -0.26 | 0.04 | -0.57 | 0.42 | -0.28 | 0.42 | 0.03 | -0.16 |
| L.MTG.middle.temporal.gyrus | -0.19 | -0.20 | -0.61 | 0.50 | -0.32 | 0.49 | 0.02 | -0.32 |
| R.OCP.occipital.pole | 0.34 | -0.10 | 0.57 | -0.48 | -0.32 | 0.42 | -0.02 | -0.03 |
| L.OCP.occipital.pole | 0.49 | 0.06 | 0.37 | -0.35 | -0.27 | 0.39 | -0.06 | -0.05 |
| R.OFuG.occipital.fusiform.gyrus | 0.03 | 0.06 | 0.09 | -0.18 | -0.33 | 0.68 | -0.08 | -0.31 |
| L.OFuG.occipital.fusiform.gyrus | 0.13 | 0.02 | 0.11 | -0.22 | -0.16 | 0.26 | -0.10 | -0.28 |
| R.OpIFG.opercular.part.of.the.inferior.frontal.gyrus | -0.37 | 0.33 | -0.11 | 0.37 | 0.21 | 0.13 | 0.02 | 0.11 |
| L.OpIFG.opercular.part.of.the.inferior.frontal.gyrus | -0.41 | 0.21 | -0.21 | 0.25 | 0.20 | -0.35 | -0.04 | 0.21 |
| R.OrIFG.orbital.part.of.the.inferior.frontal.gyrus | -0.22 | -0.34 | 0.35 | -0.43 | 0.15 | -0.12 | 0.03 | 0.05 |
| L.OrIFG.orbital.part.of.the.inferior.frontal.gyrus | -0.31 | -0.31 | 0.31 | -0.41 | 0.18 | -0.19 | -0.10 | 0.14 |
| R.PCgG.posterior.cingulate.gyrus | 0.42 | 0.22 | 0.03 | -0.04 | -0.29 | 0.70 | 0.09 | -0.13 |
| L.PCgG.posterior.cingulate.gyrus | 0.32 | -0.08 | -0.02 | -0.08 | -0.36 | 0.59 | -0.04 | 0.01 |
| R.PCu.precuneus | 0.14 | 0.24 | 0.04 | 0.08 | -0.41 | 0.40 | 0.05 | -0.18 |
| L.PCu.precuneus | 0.15 | 0.14 | 0.09 | -0.12 | -0.61 | 0.55 | -0.03 | -0.04 |
| R.PHG.parahippocampal.gyrus | 0.33 | 0.50 | 0.41 | -0.02 | 0.14 | 0.44 | -0.01 | -0.09 |
| L.PHG.parahippocampal.gyrus | 0.22 | 0.13 | 0.16 | -0.03 | -0.02 | 0.26 | -0.24 | -0.12 |
| R.PIns.posterior.insula | -0.15 | 0.31 | 0.74 | -0.31 | 0.59 | -0.44 | 0.27 | 0.07 |
| L.PIns.posterior.insula | -0.04 | 0.15 | 0.85 | -0.39 | 0.69 | -0.37 | 0.39 | 0.05 |
| R.PO.parietal.operculum | 0.00 | 0.23 | 0.66 | -0.22 | 0.12 | 0.16 | 0.05 | 0.15 |
| L.PO.parietal.operculum | -0.14 | 0.04 | 1.07 | -0.43 | 0.48 | -0.04 | 0.00 | 0.23 |
| R.PoG.postcentral.gyrus | 0.47 | -0.25 | -0.41 | 0.08 | -0.19 | 0.09 | -0.05 | -0.05 |
| L.PoG.postcentral.gyrus | 0.30 | -0.39 | -0.53 | 0.11 | 0.04 | -0.40 | -0.14 | -0.07 |
| R.POrG.posterior.orbital.gyrus | 0.08 | -0.11 | 0.69 | -0.42 | 0.25 | 0.26 | 0.05 | 0.14 |
| L.POrG.posterior.orbital.gyrus | -0.04 | 0.13 | 0.60 | -0.41 | 0.22 | -0.02 | -0.04 | 0.22 |
| R.PP.planum.polare | -0.14 | 0.20 | 0.39 | -0.07 | 0.11 | 0.12 | 0.22 | -0.11 |
| L.PP.planum.polare | -0.19 | -0.10 | 0.55 | -0.19 | -0.01 | 0.43 | 0.12 | 0.07 |
| R.PrG.precentral.gyrus | -0.44 | -0.28 | -0.66 | 0.43 | 0.09 | -0.21 | -0.17 | -0.16 |
| L.PrG.precentral.gyrus | -0.33 | -0.04 | -0.58 | 0.29 | 0.34 | -0.33 | -0.03 | -0.05 |
| R.PT.planum.temporale | -0.02 | 0.26 | 0.62 | -0.15 | 0.06 | 0.34 | 0.17 | 0.02 |
| L.PT.planum.temporale | -0.26 | -0.14 | 1.28 | -0.43 | 0.52 | 0.02 | 0.11 | 0.14 |
| R.SCA.subcallosal.area | -0.57 | 0.70 | 0.23 | -0.10 | 0.57 | -0.26 | 0.27 | 0.00 |
| L.SCA.subcallosal.area | -0.73 | 0.68 | 0.28 | -0.03 | 0.67 | -0.53 | 0.23 | -0.02 |
| R.SFG.superior.frontal.gyrus | -0.73 | 0.64 | -0.45 | 0.20 | 0.39 | -0.39 | 0.15 | -0.25 |
| L.SFG.superior.frontal.gyrus | -0.72 | 0.46 | -0.26 | 0.05 | 0.35 | -0.36 | 0.26 | -0.18 |
| R.SMC.supplementary.motor.cortex | -0.70 | 0.43 | -0.67 | 0.39 | -0.37 | 0.26 | -0.02 | 0.01 |
| L.SMC.supplementary.motor.cortex | -0.65 | 0.65 | -0.58 | 0.28 | -0.42 | 0.34 | 0.00 | 0.06 |
| R.SMG.supramarginal.gyrus | -0.05 | -0.23 | -0.40 | 0.16 | -0.04 | -0.05 | -0.22 | -0.12 |
| L.SMG.supramarginal.gyrus | 0.00 | 0.12 | -0.31 | 0.00 | 0.09 | -0.17 | -0.27 | -0.02 |
| R.SOG.superior.occipital.gyrus | 0.11 | -0.21 | 0.15 | -0.31 | -0.27 | 0.24 | -0.09 | -0.25 |
| L.SOG.superior.occipital.gyrus | 0.24 | -0.07 | -0.02 | -0.21 | -0.25 | 0.07 | -0.18 | -0.08 |
| R.SPL.superior.parietal.lobule | 0.09 | -0.20 | -0.15 | -0.11 | -0.08 | -0.10 | -0.09 | 0.00 |
| L.SPL.superior.parietal.lobule | 0.19 | -0.35 | -0.19 | 0.03 | -0.25 | 0.17 | -0.02 | -0.02 |
| R.STG.superior.temporal.gyrus | 0.02 | 0.36 | -0.63 | 0.23 | -0.14 | 0.37 | 0.00 | -0.16 |
| L.STG.superior.temporal.gyrus | -0.12 | 0.27 | -0.67 | 0.29 | -0.18 | 0.50 | -0.01 | -0.16 |
| R.TMP.temporal.pole | 0.12 | -0.29 | -0.22 | 0.12 | -0.30 | 0.34 | 0.10 | -0.10 |
| L.TMP.temporal.pole | -0.15 | -0.19 | -0.06 | 0.06 | -0.37 | 0.37 | 0.01 | -0.13 |
| R.TrIFG.triangular.part.of.the.inferior.frontal.gyrus | -0.41 | 0.38 | 0.15 | -0.15 | 0.10 | 0.18 | 0.06 | 0.21 |
| L.TrIFG.triangular.part.of.the.inferior.frontal.gyrus | -0.50 | 0.35 | 0.14 | -0.26 | 0.04 | -0.14 | -0.14 | 0.29 |
| R.TTG.transverse.temporal.gyrus | -0.05 | 0.20 | 0.85 | -0.22 | 0.09 | 0.43 | 0.16 | 0.08 |
| L.TTG.transverse.temporal.gyrus | -0.27 | -0.01 | 1.37 | -0.47 | 0.53 | 0.16 | 0.13 | 0.15 |

## Supplementary Table 11: Cohen’s d values for neuromorphometric brain regions.

Legend: Cohen’s d profiles obtained from contrasts between the 8 CNV groups and controls (adjusted for age, age^2^, sex, site, and total grey matter). Whole brain was parcellated with the neuromorphometric atlas (Supplementary Method 1) and 130 grey matter regions were used to create Cohen’s d profiles.

|  | **ROI** | **PC1** |  | **ROI** | **PC2** |
| --- | --- | --- | --- | --- | --- |
| 1. | Left_Cerebellum_Exterior | -3.186 |  | Left_Thalamus_Proper | -3.268 |
| 2. | Right_Cerebellum_Exterior | -2.992 |  | Right_Thalamus_Proper | -2.988 |
| 3. | Left_middle_cingulate_gyrus | -2.808 |  | Left_Ventral_DC | -2.800 |
| 4. | Left_fusiform_gyrus | -2.666 |  | Right_Ventral_DC | -2.616 |
| 5. | Right_middle_cingulate_gyrus | -2.664 |  | Left_calcarine_cortex | -2.563 |
| 6. | Left_middle_temporal_gyrus | -2.608 |  | Right_occipital_pole | -2.426 |
| 7. | Left_inferior_temporal_gyrus | -2.473 |  | Right_calcarine_cortex | -2.308 |
| 8. | Right_fusiform_gyrus | -2.215 |  | Cerebellar_Vermal_Lobules_VIII-X | -2.108 |
| 9. | Left_cuneus | -2.152 |  | Right_Pallidum | -2.031 |
| 10. | Left_angular_gyrus | -2.120 |  | Left_Pallidum | -2.030 |
| 11. | Left_Accumbens_Area | 2.487 |  | Right_frontal_pole | 2.392 |
| 12. | Right_subcallosal_area | 2.617 |  | Left_superior_frontal_gyrus | 2.443 |
| 13. | Left_parietal_operculum | 2.646 |  | Left_superior_frontal_gyrus_medial_segment | 2.459 |
| 14. | Left_planum_temporale | 2.838 |  | Left_supplementary_motor_cortex | 2.487 |
| 15. | Left_transverse_temporal_gyrus | 2.876 |  | Right_supplementary_motor_cortex | 2.626 |
| 16. | Left_subcallosal_area | 3.022 |  | Left_middle_cingulate_gyrus | 2.734 |
| 17. | Right_posterio_.insula | 3.315 |  | Right_superior_frontal_gyrus_medial_segment | 2.862 |
| 18. | Left_anterior_insula | 3.654 |  | Right_middle_frontal_gyrus | 3.073 |
| 19. | Left_posterior_insula | 3.666 |  | Right_superior_frontal_gyrus | 3.099 |
| 20. | Right_anterior_insula | 3.784 |  | Left_middle_frontal_gyrus | 3.302 |

## Supplementary Table 12: Principal Component Analysis ROIs loadings (volume)

Legend: Top 20 loadings (10 negative, 10 positive) of Neuromorphometric Regions of Interests (ROIs) on the 2 PCA dimensions (the first 2 components explained respectively 31.77 and 28.66 % of the variance). Loadings values are listed in increasing order, top 10 negative followed by top 10 positive.

|  | **ROI** | **\|CC1\|** |  | **ROI** | **\|CC2\|** |
| --- | --- | --- | --- | --- | --- |
| 1. | Left_transverse_temporal_gyrus | 0.54424372 |  | Right_Accumbens_Area | 0.716341907 |
| 2. | Left_planum_temporale | 0.516375486 |  | Left_Accumbens_Area | 0.693450348 |
| 3. | Left_parietal_operculum | 0.478605816 |  | Left_subcallosal_area | 0.598630484 |
| 4. | Left_calcarine_cortex | 0.434979442 |  | Left_cuneus | 0.587180378 |
| 5. | Right_supplementary_motor_cortex | 0.423161744 |  | Left_precuneus | 0.542742079 |
| 6. | Right_calcarine_cortex | 0.417872614 |  | Brain_Stem | 0.538584491 |
| 7. | Left_posterior_insula | 0.41658809 |  | Right_superior_frontal_gyrus | 0.530594854 |
| 8. | Left_middle_cingulate_gyrus | 0.404137535 |  | Cerebellar_Vermal_Lobules_VIII-X | 0.530148089 |
| 9. | Left_middle_temporal_gyrus | 0.380330333 |  | Left_anterior_insula | 0.527867312 |
| 10. | Right_anterior_insula | 0.378170585 |  | Right_lingual_gyrus | 0.526385942 |
| 11. | Right_posterior_orbital_gyrus | 0.375392577 |  | Left_lingual_gyrus | 0.52497161 |
| 12. | Left_anterior_insula | 0.37348146 |  | Right_cuneus | 0.513604455 |
| 13. | Right_occipital_pole | 0.367616101 |  | Right_Caudate | 0.502454679 |
| 14. | Left_supplementary_motor_cortex | 0.35555794 |  | Right_anterior_insula | 0.49874064 |
| 15. | Right_precentral_gyrus | 0.354152555 |  | Left_calcarine_cortex | 0.486781547 |
| 16. | Right_posterior_insula | 0.352835632 |  | Left_superior_frontal_gyrus | 0.481515906 |
| 17. | Right_middle_temporal_gyrus | 0.351133642 |  | Left_Cerebellum_Exterior | 0.477760835 |
| 18. | Right_middle_cingulate_gyrus | 0.343736614 |  | Left_gyrus_rectus | 0.468197071 |
| 19. | Right_middle_frontal_gyrus | 0.341059273 |  | Right_subcallosal_area | 0.465928639 |
| 20. | Left_Thalamus_Proper | 0.340754975 |  | Right_calcarine_cortex | 0.463409682 |

## Supplementary Table 13: Canonical Correlation Analysis ROIs loadings (volume)

Legend: Top 20 loadings of Neuromorphometric Regions of Interests (ROIs) on the 2 main CCA dimensions (r=0.84, 0.79; statistically significant at p-value<0.05). Loadings are in absolute values and listed in decreasing order.

|  | **ROI** | **\|CC1\|** |  | **ROI** | **\|CC2\|** |
| --- | --- | --- | --- | --- | --- |
| 1. | L_superiortemporal | 0.6587446385 |  | R_supramarginal | 0.4556194543 |
| 2. | R_superiortemporal | 0.4716305815 |  | R_postcentral | 0.4078724885 |
| 3. | R_pericalcarine | 0.3752495588 |  | L_caudalanteriorcingulate | 0.3529766412 |
| 4. | L_bankssts | 0.3625881379 |  | L_postcentral | 0.3515379435 |
| 5. | L_transversetemporal | 0.3385850519 |  | R_pericalcarine | 0.3243996464 |
| 6. | L_insula | 0.3145652172 |  | L_inferiortemporal | 0.3037588837 |
| 7. | R_caudalanteriorcingulate | 0.3088200827 |  | R_inferiortemporal | 0.2836174352 |
| 8. | L_parahippocampal | 0.3074088432 |  | L_parstriangularis | 0.2467471024 |
| 9. | L_caudalanteriorcingulate | 0.2822321503 |  | R_superiortemporal | 0.2382066534 |
| 10. | L_lingual | 0.2806560547 |  | L_fusiform | 0.2357128834 |
| 11. | L_pericalcarine | 0.2775258853 |  | L_lingual | 0.2303667725 |
| 12. | L_inferiorparietal | 0.249919579 |  | R_fusiform | 0.2224770313 |
| 13. | R_insula | 0.243533976 |  | L_pericalcarine | 0.2217702203 |
| 14. | L_inferiortemporal | 0.2406929256 |  | R_lateraloccipital | 0.2163886563 |
| 15. | R_lingual | 0.2355838232 |  | L_cuneus | 0.2143733739 |
| 16. | R_posteriorcingulate | 0.2275119787 |  | R_superiorparietal | 0.2064224327 |
| 17. | L_fusiform | 0.2234845007 |  | L_supramarginal | 0.2042270199 |
| 18. | R_bankssts | 0.2160471626 |  | L_lateralorbitofrontal | 0.200380036 |
| 19. | L_superiorparietal | 0.213339904 |  | L_lateraloccipital | 0.1888223609 |
| 20. | R_cuneus | 0.2122297907 |  | R_rostralanteriorcingulate | 0.1811606572 |

## Supplementary Table 14: Canonical Correlation Analysis ROIs loadings (CT)

Legend: Top 20 loadings of Desikan Regions of Interests (ROIs) on the 2 main CCA dimensions (r=0.69, 0.65; statistically significant at p-value<0.05). Loadings are in absolute values and listed in decreasing order.

|  | **ROI** | **\|CC1\|** |  | **ROI** | **\|CC2\|** |
| --- | --- | --- | --- | --- | --- |
| 1. | L_bankssts | 0.6521173442 |  | L_cuneus | 0.4721872379 |
| 2. | L_lateraloccipital | 0.6217516421 |  | R_precentral | 0.4287453486 |
| 3. | L_insula | 0.5772778171 |  | L_caudalmiddlefrontal | 0.3853658259 |
| 4. | R_bankssts | 0.5730431727 |  | L_lingual | 0.3836281323 |
| 5. | R_lateraloccipital | 0.5575884583 |  | L_precentral | 0.3658962124 |
| 6. | R_insula | 0.5274745577 |  | R_lingual | 0.3579919796 |
| 7. | R_lingual | 0.4881908917 |  | R_superiorfrontal | 0.3560307613 |
| 8. | L_pericalcarine | 0.4619077081 |  | L_lateralorbitofrontal | 0.3541644729 |
| 9. | L_lateralorbitofrontal | 0.443217953 |  | L_insula | 0.3306590802 |
| 10. | L_entorhinal | 0.4227964373 |  | R_insula | 0.3119737927 |
| 11. | R_pericalcarine | 0.4101667004 |  | L_precuneus | 0.3104848234 |
| 12. | L_lingual | 0.4071596452 |  | R_cuneus | 0.3006340465 |
| 13. | R_parsorbitalis | 0.4015238848 |  | R_precuneus | 0.2940527986 |
| 14. | L_postcentral | 0.3973332167 |  | L_superiorparietal | 0.2856777808 |
| 15. | L_caudalmiddlefrontal | 0.3914227421 |  | R_lateralorbitofrontal | 0.2854749482 |
| 16. | L_middletemporal | 0.3882833195 |  | R_superiorparietal | 0.2735339712 |
| 17. | R_middletemporal | 0.3879412723 |  | L_superiorfrontal | 0.271298681 |
| 18. | R_caudalmiddlefrontal | 0.3821714147 |  | L_pericalcarine | 0.2670122335 |
| 19. | L_inferiortemporal | 0.379691104 |  | R_parsopercularis | 0.2515318479 |
| 20. | R_superiorfrontal | 0.3789751734 |  | L_postcentral | 0.2398170347 |

## Supplementary Table 15: Canonical Correlation Analysis ROIs loadings (SA)

Legend: Top 20 loadings of Desikan Regions of Interests (ROIs) on the 2 main CCA dimensions (r=0.79, 0.67; statistically significant at p-value<0.05). Loadings are in absolute values and listed in decreasing order.


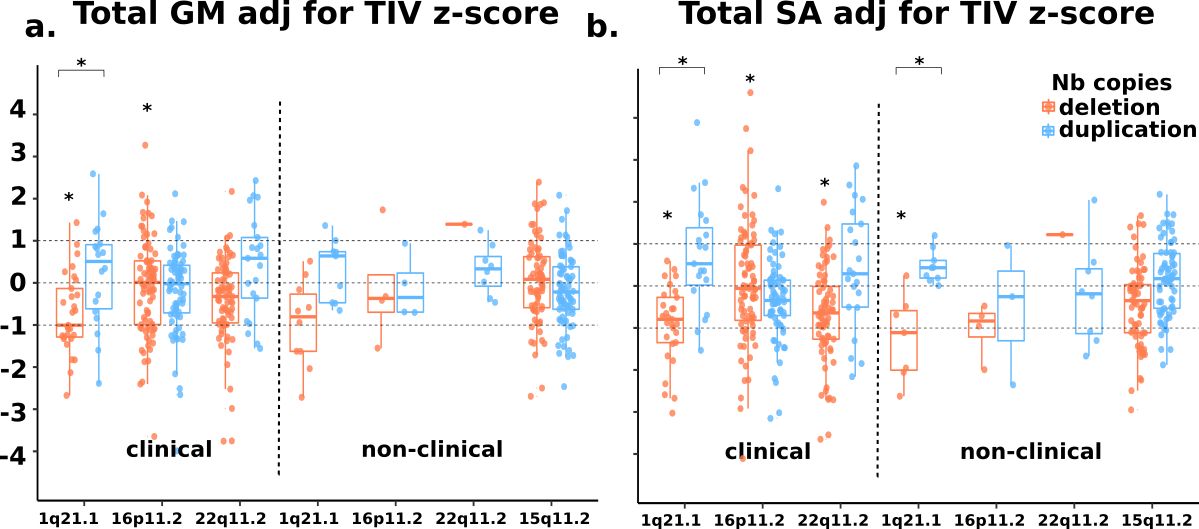


## Supplementary Figure 1: The effect of 1q21.1, 16p11.2, 22q11.2 and 15q11.2 on total GM and total SA adjusted for TIV

Legend: Total grey matter volume (a) and total surface area (b) for clinically and non-clinically ascertained CNVs. Z-scores for clinically and non-clinically ascertained CNVs were calculated using 331 and 965 controls respectively, adjusting for age, age^2^, sex, TIV and site as a random factor. Y axis values are z scores. X axis are CNV groups. Significant difference between CNV group and corresponding control group is indicated with a star. Horizontal bars with stars show significant differences between deletions and duplications within the same locus.

*
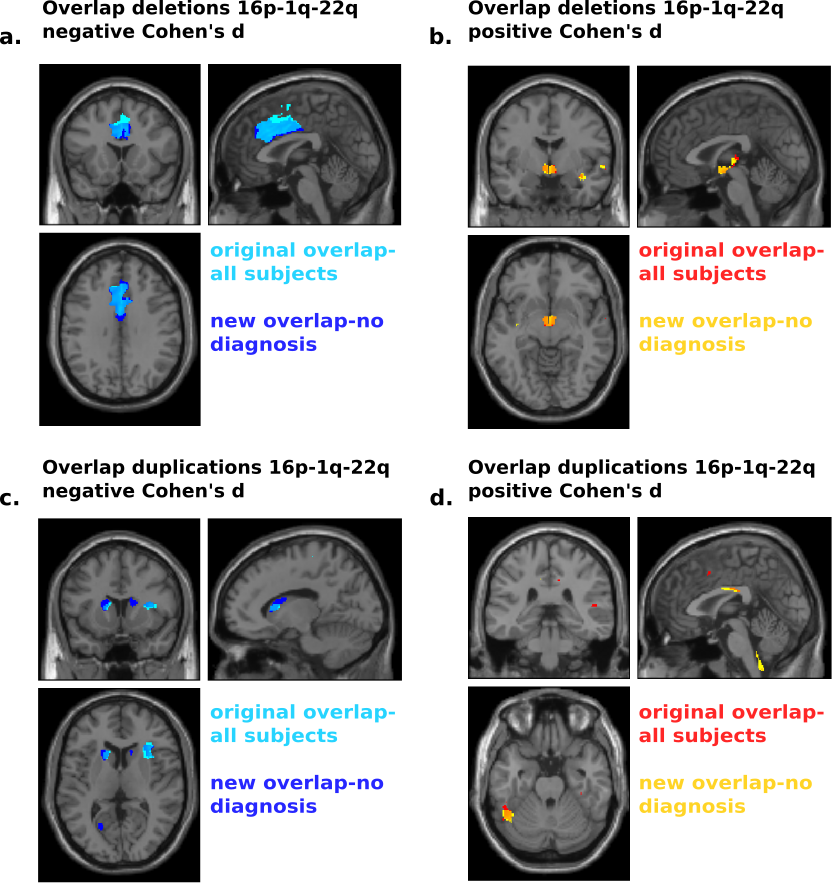
*

## Supplementary Figure 2: Robustness of spatial overlap after removing subjects with psychiatric diagnosis

Legend: Comparison of spatial overlap between the grey matter volume effects of 3 clinically ascertained CNVs 1q21.1, 16p11.2 and 22q11.2 obtained from contrast controls including and excluding subjects with diagnosis. Overlap comparison of (a) negative effects and (b) positive effects for deletions. Overlap comparison of (c) negative effects and (d) positive effects for duplications. Original overlaps calculated from contrasts including all subjects are shown in cyan (a,c) and red (b,d). New overlaps calculated from contrast excluding subjects with a psychiatric diagnosis are shown in blue (a,c) and yellow (b,d).


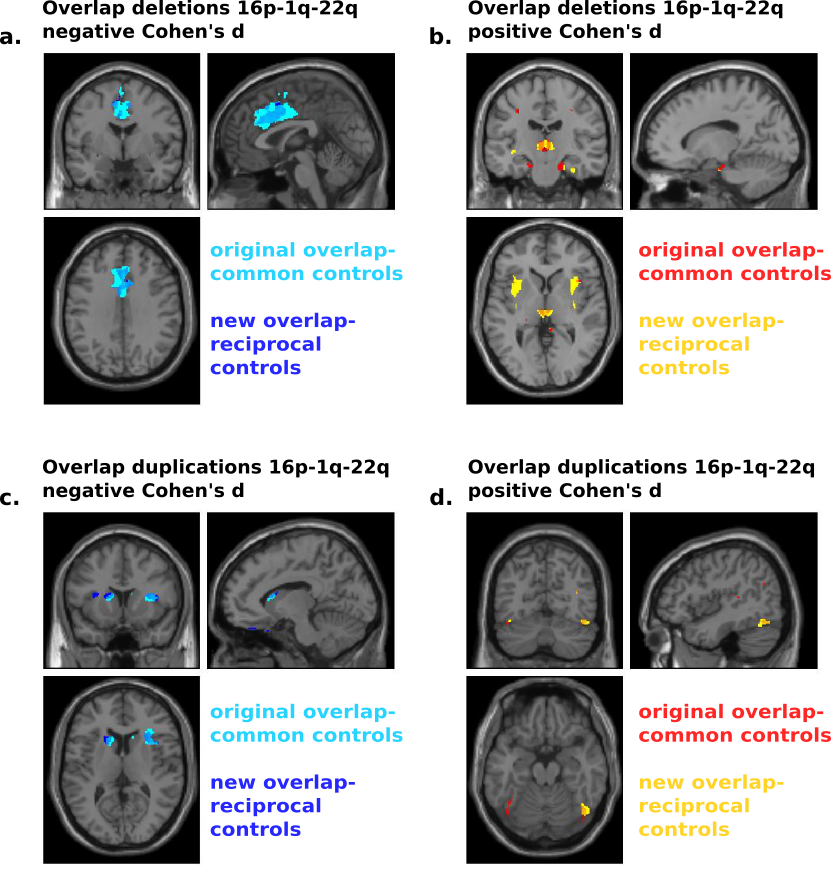


## Supplementary Figure 3: Robustness of spatial overlap after matching controls by site

Legend: Comparison of spatial overlap between the grey matter volume effects of 3 clinically ascertained CNVs 1q21.1, 16p11.2 and 22q11.2 obtained from contrast with merged or contrast with separated reciprocal controls. Overlap comparison of (a) negative effects and (b) positive effects for deletions. Overlap comparison of (c) negative effects and (d) positive effects for duplications. Original overlaps calculated from contrasts with the merged group of controls are shown in cyan (a,c) and red (b,d). New overlaps calculated from contrast with reciprocal control groups for each CNV are shown in blue (a,c) and yellow (b,d).

##
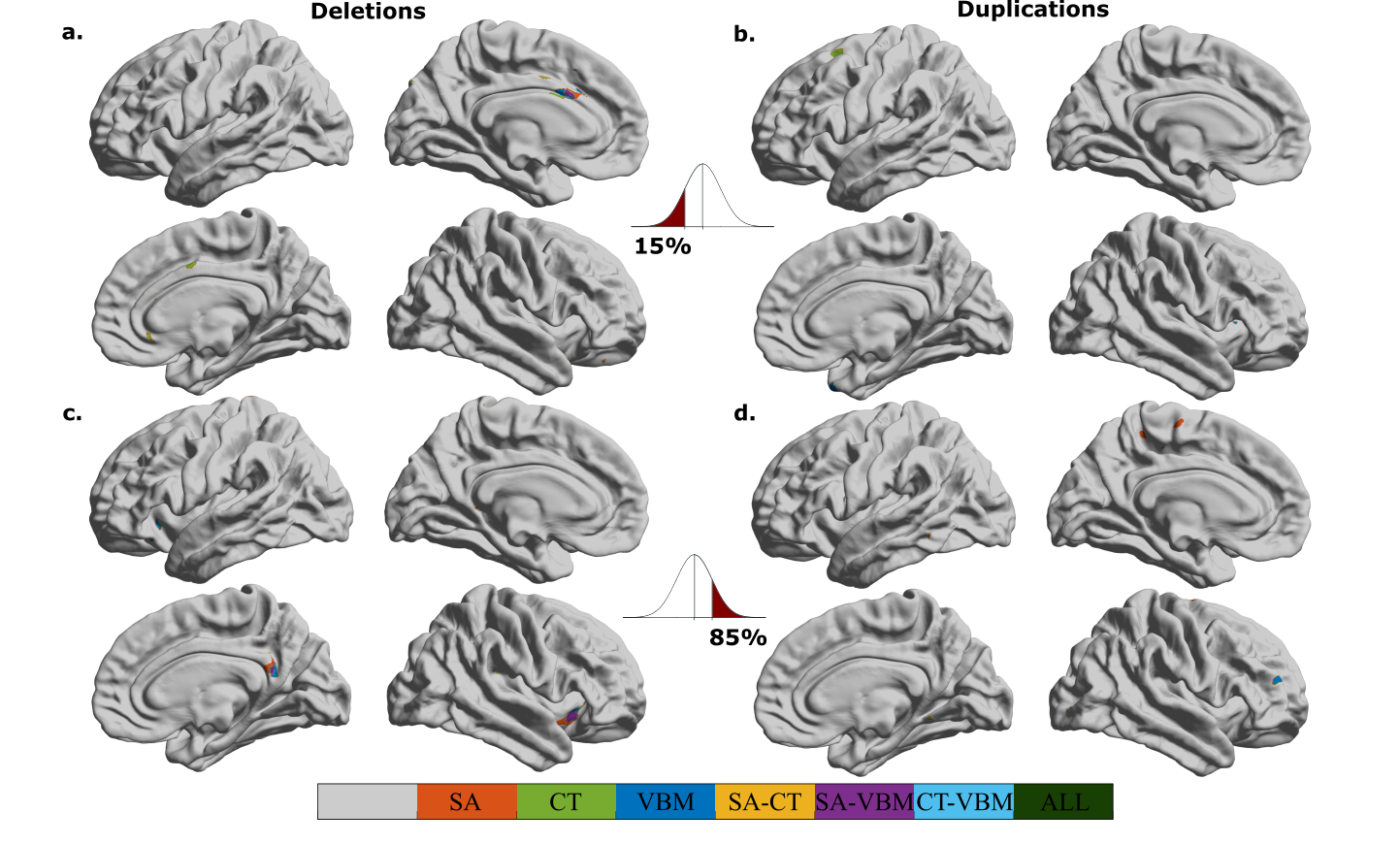
 Supplementary Figure 4: Intersection of deletion and duplication overlap maps of 4 CNVs across SA-CT-VBM

Legend: Deletion (left) and duplication (right) overlap maps for 1q21.1, 15q11.2, 16p11.2, and 22q11.2 and intersection between pairs of SA-CT-VBM overlap maps for both tails <15% (top row) and >85% (bottom row). For comparison, VBM overlap maps are projected onto fsaverage using mri_vol2surf function in Freesurfer. See Supplementary Table 15 for a summary of the number of intersecting vertices within Desikan ROIs between pairs of SA-CT-VBM. More intersection is observed for SA-VBM (purple) as opposed to CT-VBM or SA-CT, mainly in insula, isthmus cingulate, and lateral orbitofrontal. Only 7 vertices intersect (in caudal anterior cingulate) across all three - SA-CT-VBM deletion overlap maps - for tail 1 (<15%). No intersection between pairs of SA-CT-VBM overlap maps is observed for duplications. SA: Surface Area; CT: Cortical Thickness; VBM: Voxel Based Morphometry;

##
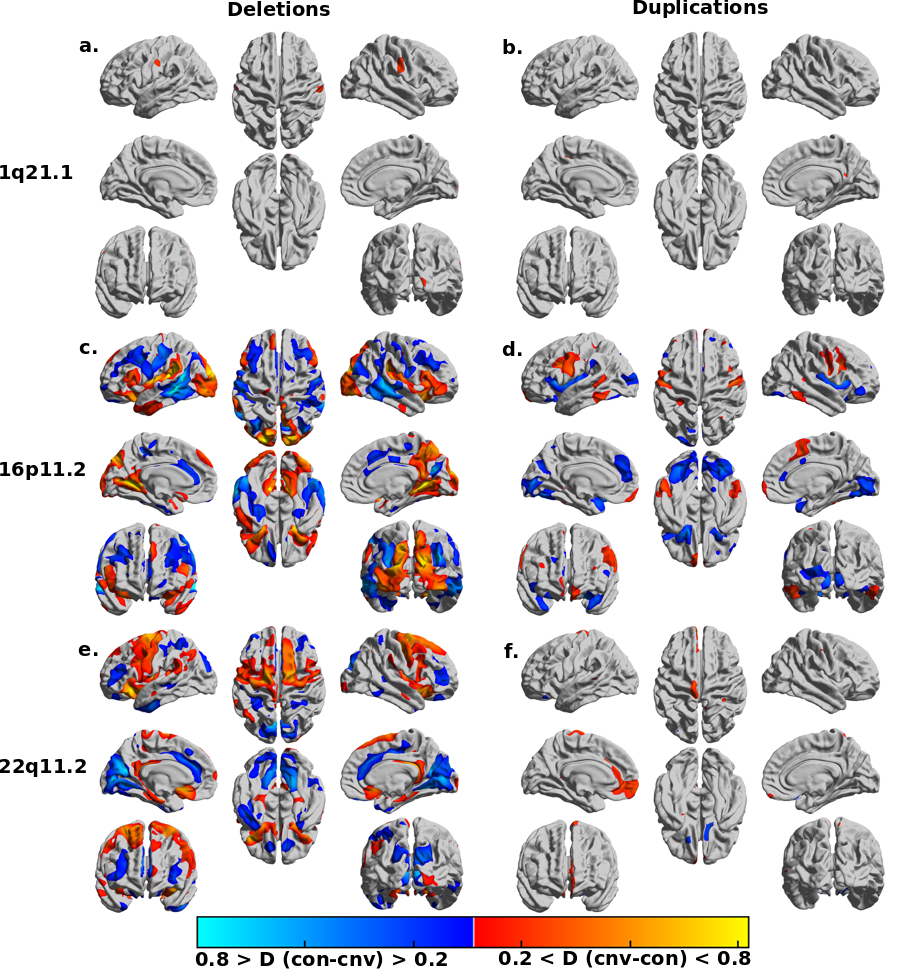


## Supplementary Figure 5: FDR corrected Cohen’s d maps of Surface Area

Legend: Vertex-wise brain differences in deletion and duplication carriers at the 1q21.1, 16p11.2 and 22q11.2. loci. 15q11.2 was not displayed because few vertices survived FDR correction. The left column shows regional brain differences for deletion carriers (a, c, e) while right columns show regional brain differences for duplication carriers (b, d, f). Cohen’s d effect sizes were estimated in SurfStat using vertex-wise SA estimate maps obtained from Freesurfer. Linear model was adjusted for sex, linear and quadratic expansion of age and total surface area. The results are significant at the FDR threshold q < 0.05, and shown within a range of 0.2 and 0.8 for comparison across CNVs.

##
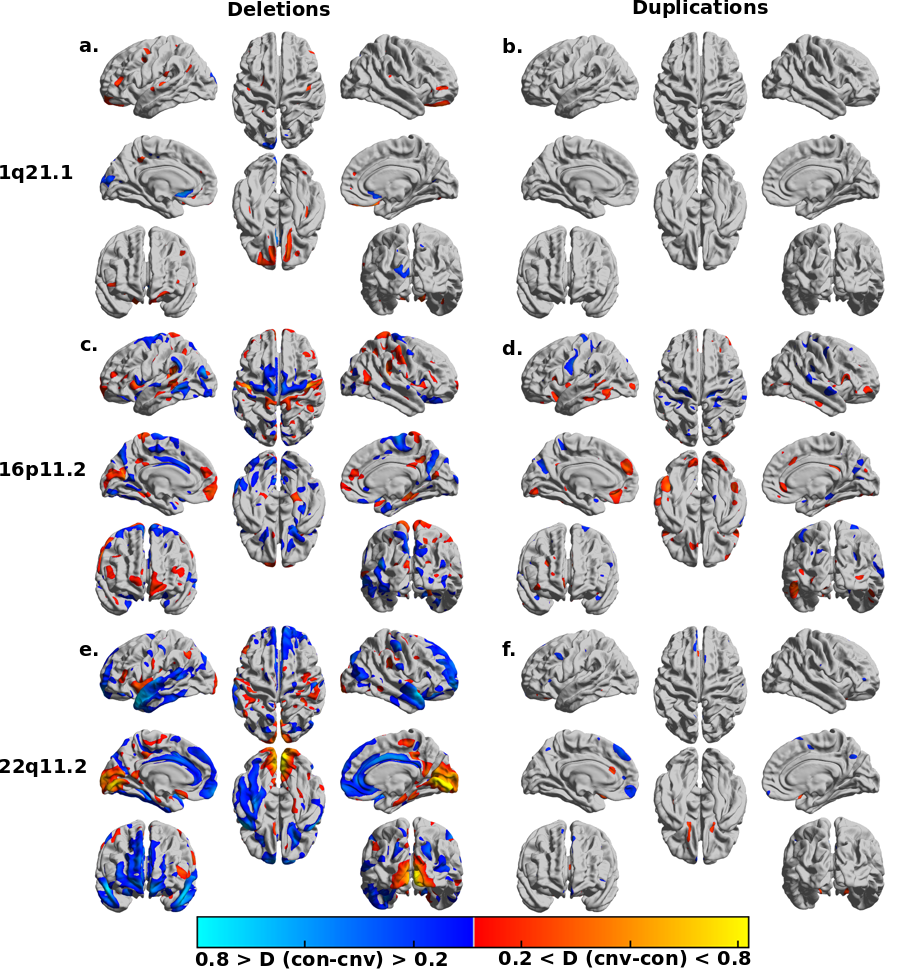


## Supplementary Figure 6: FDR corrected Cohen’s d maps of Cortical Thickness

Legend: Vertex-wise brain differences in deletion and duplication carriers at the 1q21.1, 16p11.2 and 22q11.2. loci. 15q11.2 was not displayed because few vertices survived FDR correction. Left column shows vertex-wise brain differences for deletion carriers (a, c, e) while right columns show vertex-wise brain differences for duplication carriers (b, d, f). Cohen’s d effect sizes were estimated in SurfStat using vertex-wise CT estimate maps obtained from Freesurfer. Linear model was adjusted for sex, linear and quadratic expansion of age and mean cortical thickness. The results are significant at the FDR threshold q < 0.05 and shown within a range of 0.2 and 0.8 for comparison across CNVs.


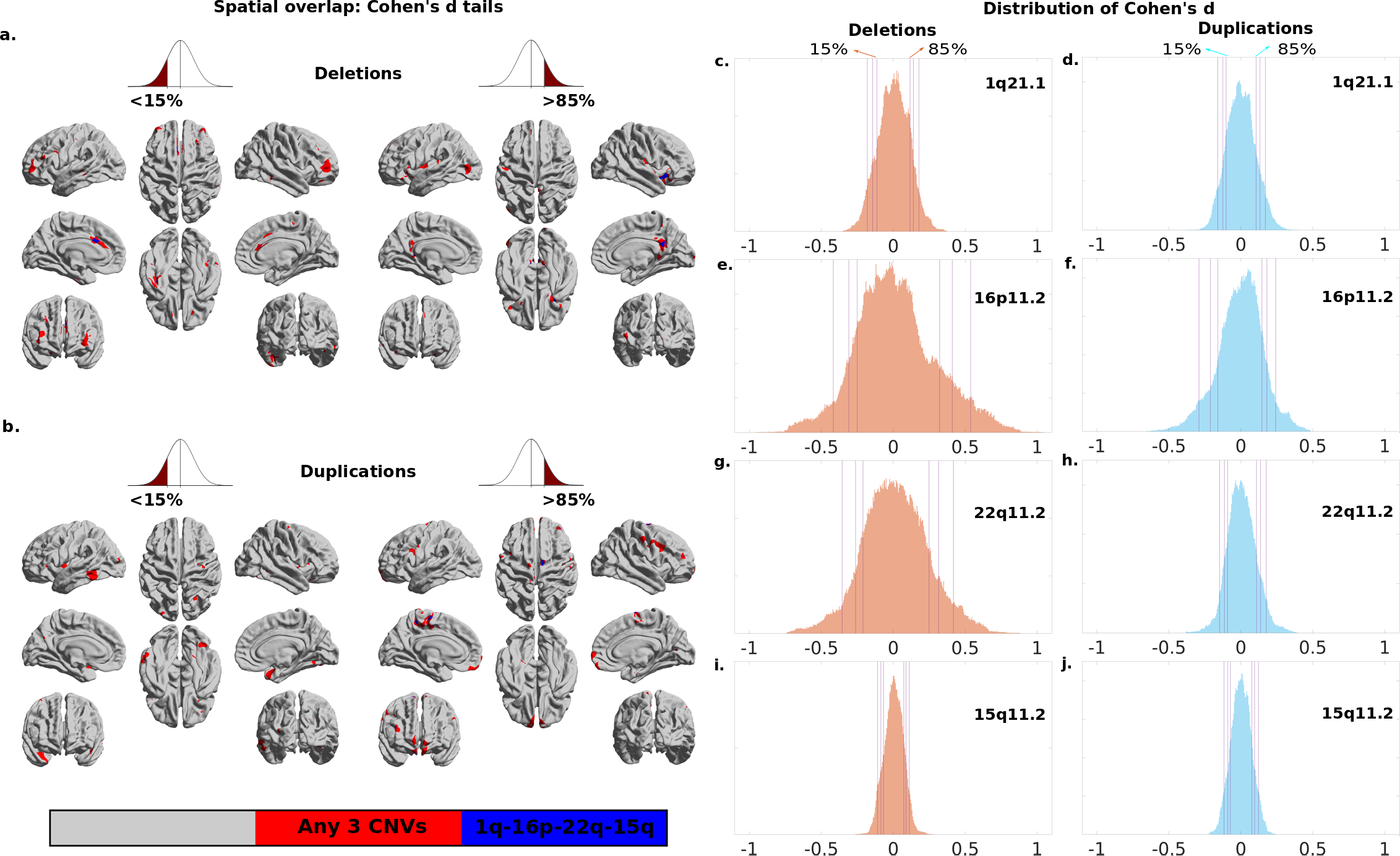


## Supplementary Figure 7: Spatial overlap for surface area (SA) across deletions and duplications

Legend: Spatial overlap across clinically and non-clinically ascertained deletions (a) and duplications (b) at 4 genomic loci shown separately for <15th and >85th percentile of Cohen’s d values. Overlap of all four deletions (a) or all four duplications (b) is shown in blue. Overlaps of any combination of three deletions (a) or any combination of three duplications (b) are shown in red. Top ranking Cohen’s d values used in (a, b) are presented on the density plots for all eight deletions and duplications: 1q21.1 (c, d), 16p11.2 (e, f), 22q11.2 (g, h), and 15q11.2 (i, j). The X axes values of the 8 density plots are Cohen’s d.

##
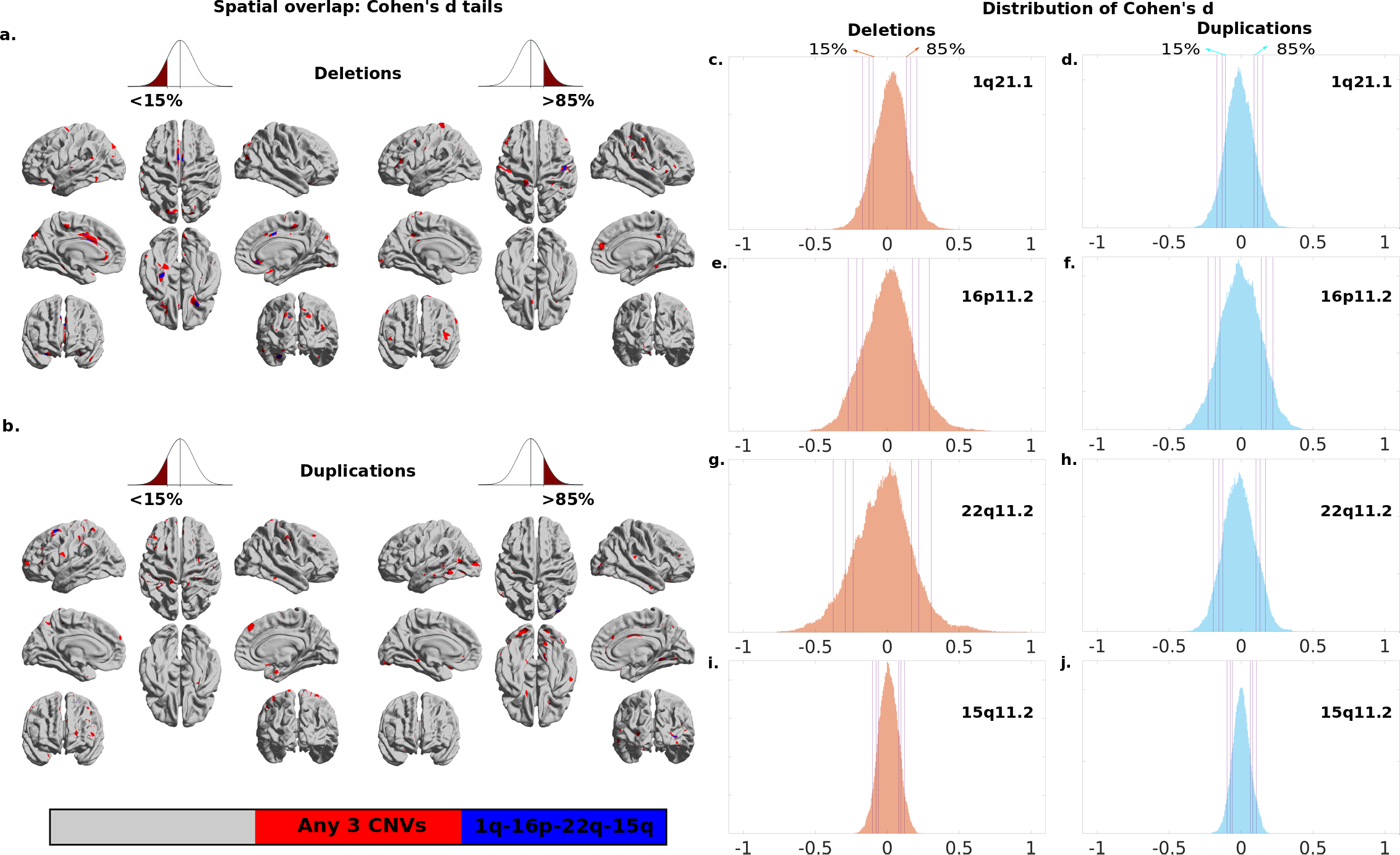


## Supplementary Figure 8: Spatial overlap for mean cortical thickness (CT) across deletions

Legend: Spatial overlap across clinically and non-clinically ascertained deletions (a) and duplications (b) at 4 genomic loci shown separately for <15th and >85th percentile of Cohen’s d values. Overlap of all four deletions (a) or all four duplications (b) is shown in blue. Overlaps of any combination of three deletions (a) or any combination of three duplications (b) are shown in red. Top ranking Cohen’s d values used in (a, b) are presented on the density plots for all eight deletions and duplications: 1q21.1 (c, d), 16p11.2 (e, f), 22q11.2 (g, h), and 15q11.2 (i, j). The X axes values of the 8 density plots are Cohen’s d.

##
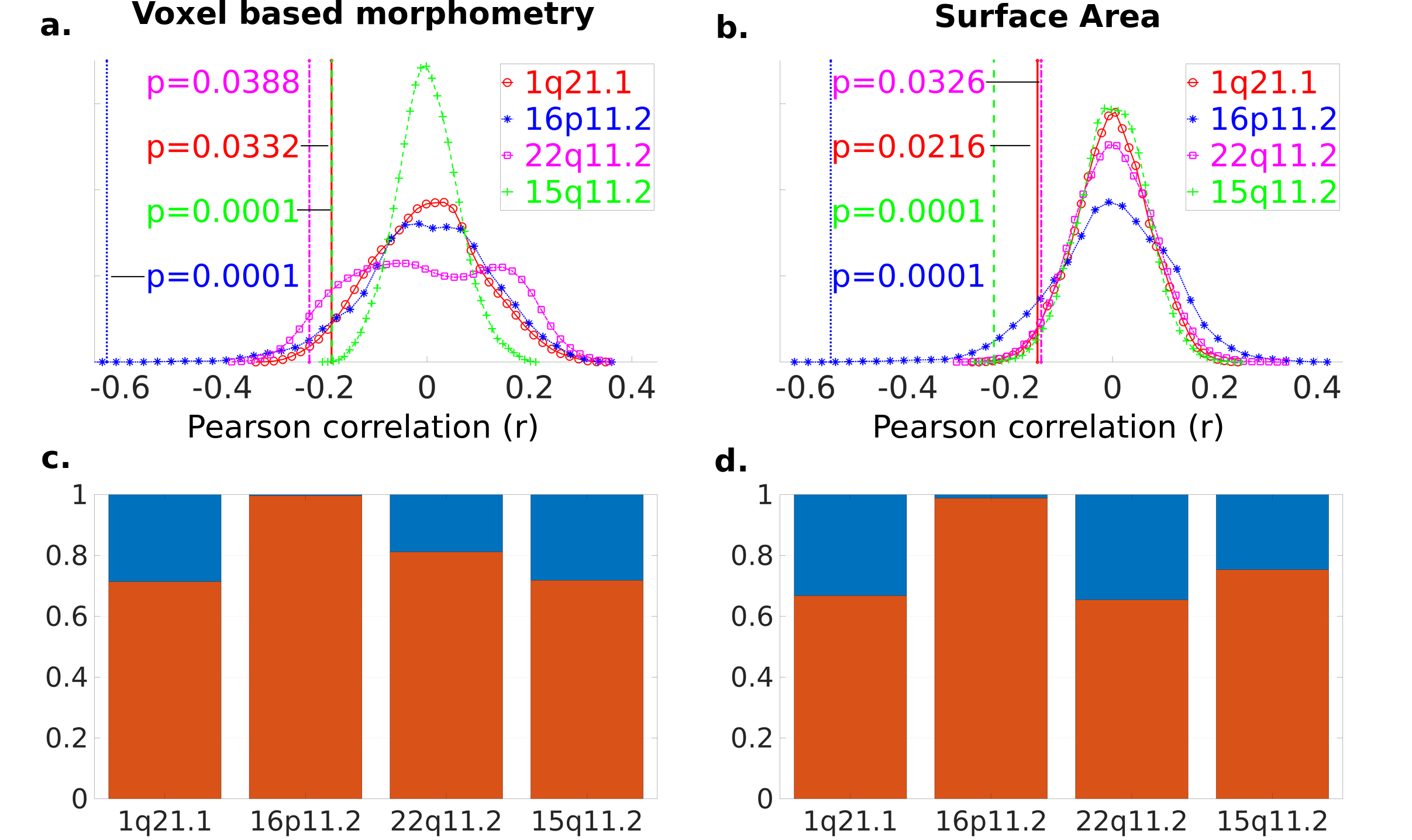


## Supplementary Figure 9: Cortex-wide mirror effects between deletions and duplications

Legend: Pearson correlations between Cohen’s d values for deletions and duplications. Voxel-Based Morphometry (a) and Surface Area (b) are adjusted for total gray matter and total surface area respectively. The 4 vertical lines represent the correlation (Pearson r) between deletions and duplications at each locus: 1q21.2 (red), 15q11.2 (green), 16p11.2 (blue), and 22q11.2 (magenta), with the corresponding empirical p-values (uncorrected) shown next to them in same color code. The 4 density plots represent the distribution of Pearson Correlations obtained by performing 10,000 spin permutations of duplication maps while keeping deletion maps fixed. Negative correlations between deletions and duplication are observed across loci and are significantly different (Bonferroni) from the null distributions for 16p11.2 and 15q11.2 (p-values are uncorrected). X axis = Pearson r coefficients, y axis = the surface under the curve is 100% of the distribution. (c-d) Mirror effects between deletions and duplications at both tails of the distribution c) The red bar is the proportion of voxels that are in the top 85th percentile for deletions and the lower 15th percentile for duplications and vice versa. The blue bar represents voxels that are either in the top 85th or lower 15th percentile for both deletions and duplications. d) The same bar plots are presented for surface area. (All Correlation values are reported in supplemental eTable 8-10).

##
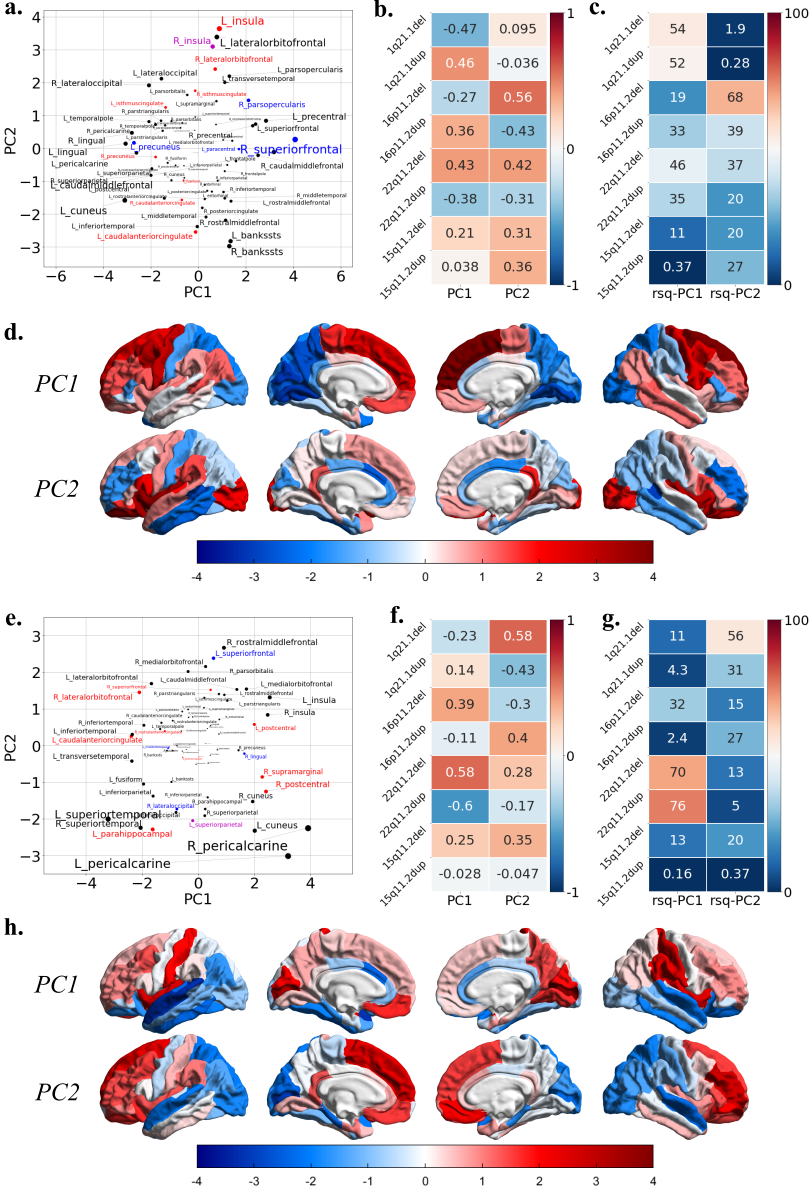
 Supplementary Figure 10: Principal Component Analysis of brain alterations associated with 8 CNVs for SA and CT

Legend: Top half (a, b, c, d) corresponds to SA, and bottom half (e, f, g, h) to CT. (a,e) Loading of ROIs on the 2 PCA dimensions; the font size is correlated to the region's contribution to dimensions. ROI names are color coded as being part of the deletion (red), duplication (blue) and both deletion and duplication (magenta) convergence maps. PCA was computed on z-scored Cohen’s d values, with the 8 CNVs as variables and 68 Desikan ROIs (FreeSurfer derived) as observations. Cohen’s d for SA/CT regions were obtained using linear models adjusted for Total-SA/Mean-CT, age, age2, sex and site. The first 2 components explained respectively 31.25 and 26.75 % of the variance for SA; and 26.13 and 20.79 % of the variance for CT. (b,f) Loading of 8 CNVs on the 2 PCA dimensions. Values are PC loading magnitudes and represent the contribution of a CNV to the PC. (c,g) Variance explained (rsq) of each CNV Cohen’s d profile by PC1 and PC2. Values and color scale represent the “percent of variance”. (d,h) PC1 and PC2 projected on the brain. The darker the red or blue color, the stronger the positive or negative loadings for PC1 and PC2.

##
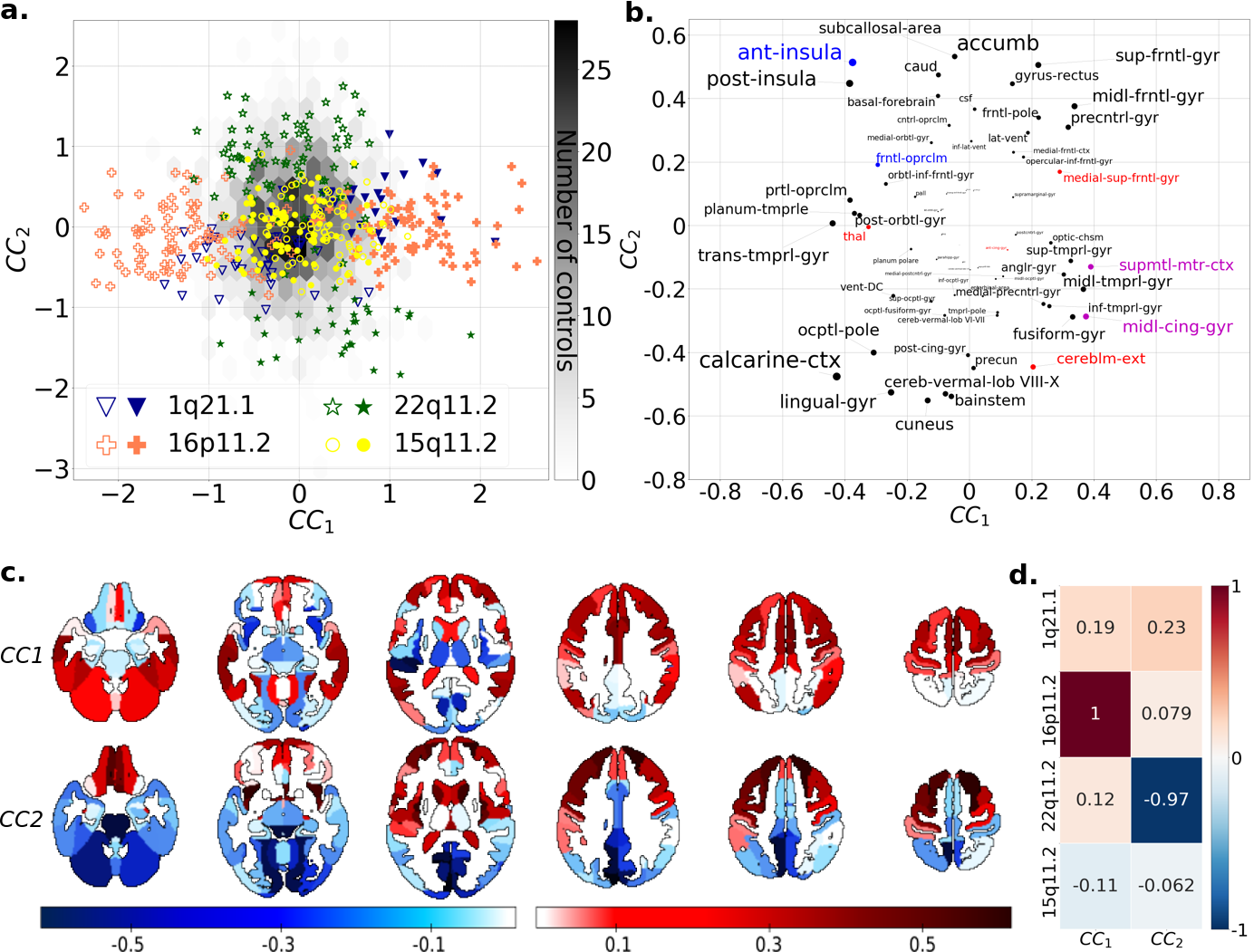
 Supplementary Figure 11: Co-analysis of shared brain alterations due to 8 CNVs (4 Genomic Loci)

Legend: Legend: (a) Scatterplot showing the position of each of the 484 carriers of 8 different CNVs along 2 dominant brain-gene Canonical Correlation (CC) dimensions established using 130 neuroanatomical GM regions of CNV carriers. GM region volumes were obtained using neuromorphometric and were adjusted for total grey matter, age, age^2^, sex and site. The empty and full symbols represent deletions and duplication respectively. The grey hexagonal bin plot represents the frequency of controls (n=1296). Controls were not used to calculate the CCA and were projected post hoc on the 2 dimensions using CCA prediction. X and Y axis values: z-scores of regional volumes. (b) Loading of Neuromorphometric Regions of Interests (ROIs) on the 2 CC dimensions. The font size is correlated to the region's contribution to dimensions. ROI names are color coded as being part of the deletion (red), duplication (blue) and both deletion and duplication (magenta) convergence patterns. (c) CCA dimension 1 and 2 regional relevances projected on axial brain slices. The darker the red or blue color, the stronger the positive or negative association with the CCA dimensions. (d) Loading of the first and second CCA dimension on 4 CNV genomic loci. Values are CCA loading magnitudes and represent the contribution of a CNV loci to the canonical dimension.

##
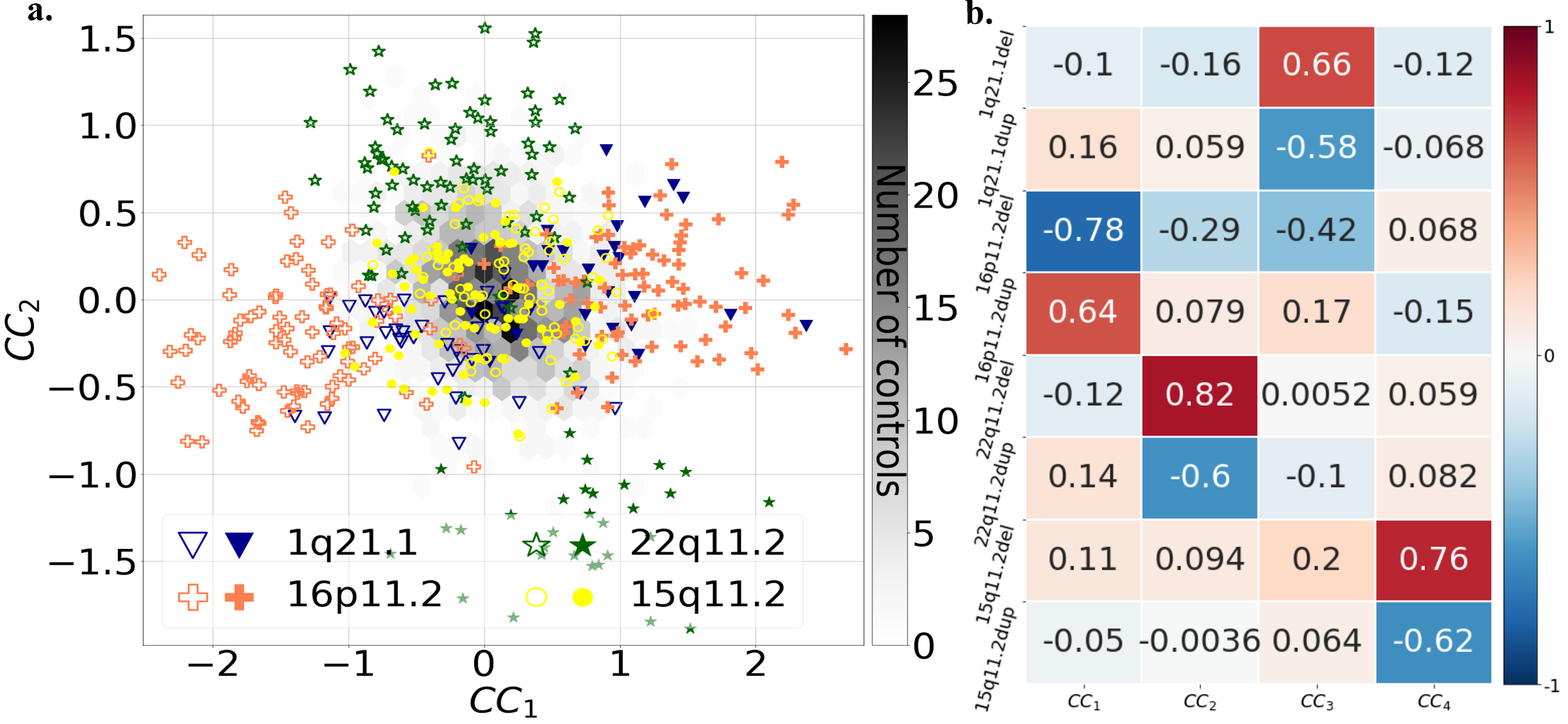
 Supplementary Figure 12: CCA analysis of 8 CNVs (Deletion and Duplication as independent groups)

Legend: (a) Scatterplot showing the position of each of the 484 carriers of 8 different CNVs along 2 Canonical Correlation (CC) dimensions established using 130 neuroanatomical GM regions of CNV carriers. GM region volumes were obtained using neuromorphometric and were adjusted for total grey matter, age, age^2^, sex and site. The empty and full symbols represent deletions and duplication respectively. The grey hexagonal bin plot represents the frequency of controls (n=1296). Controls were not used to compute the CCA and were projected post hoc on the 2 dimensions using CCA prediction. (b) Loading of the first and second CCA dimension on 8 CNVs. Values are CCA loading magnitudes and represent the contribution of a CNV to the canonical dimension.


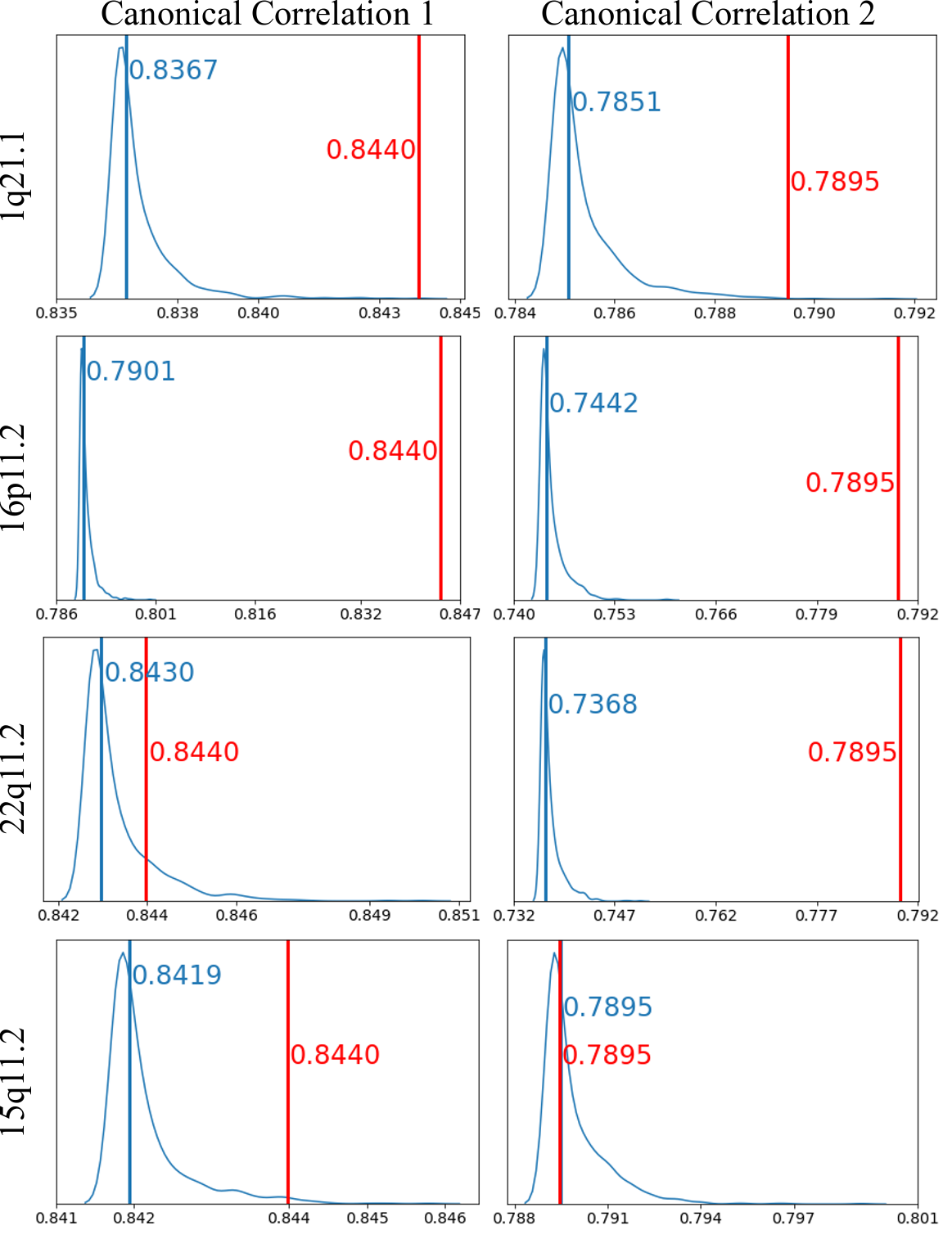


## Supplementary Figure 13: Impact of each genomic loci on Canonical Correlations

Legend: Density plots of canonical correlations (CC1: left column; CC2: right column) obtained by shuffling labels 1000 times for each genomic loci separately. The red vertical line is the original Canonical Correlation values including all genomic loci. red X-axis: Canonical Correlation. Y-axis: Frequency. Blue vertical line: median of null Canonical Correlations. We consistently observe that the original value is significantly different from the distribution of the randomly permuted values.

##
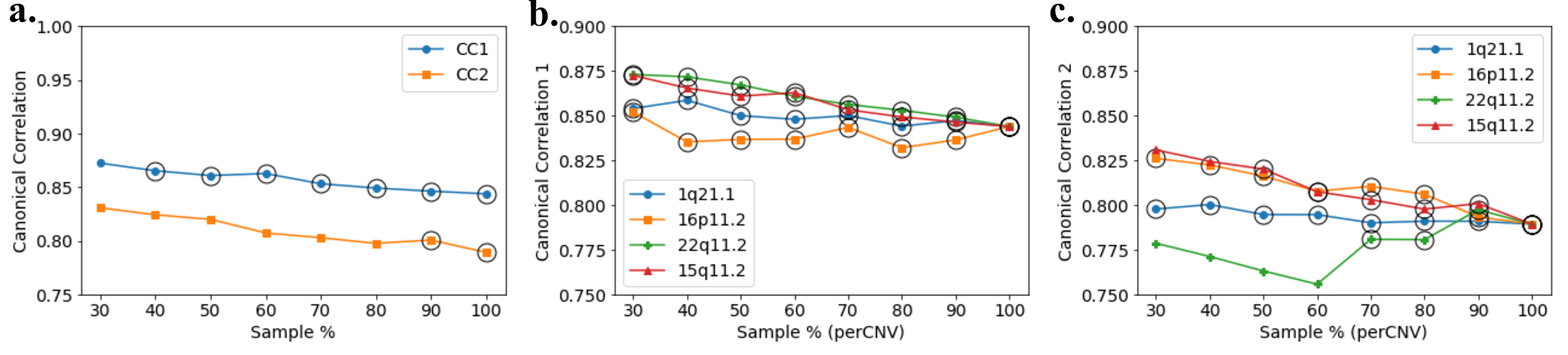


## Supplementary Figure 14: Impact of sample size on CCA analysis

Legend: (a) Plot showing the impact of sample size on Canonical Correlation 1 and 2, for all CNV carriers (4 CNV loci). X-axis: Sample %; Y-axis: Canonical Correlation. Significant CCs are encircled in black. (b-c) Plots showing the impact of sample size of single CNV loci on Canonical Correlation 1 (b), and Canonical Correlation 2 (c).


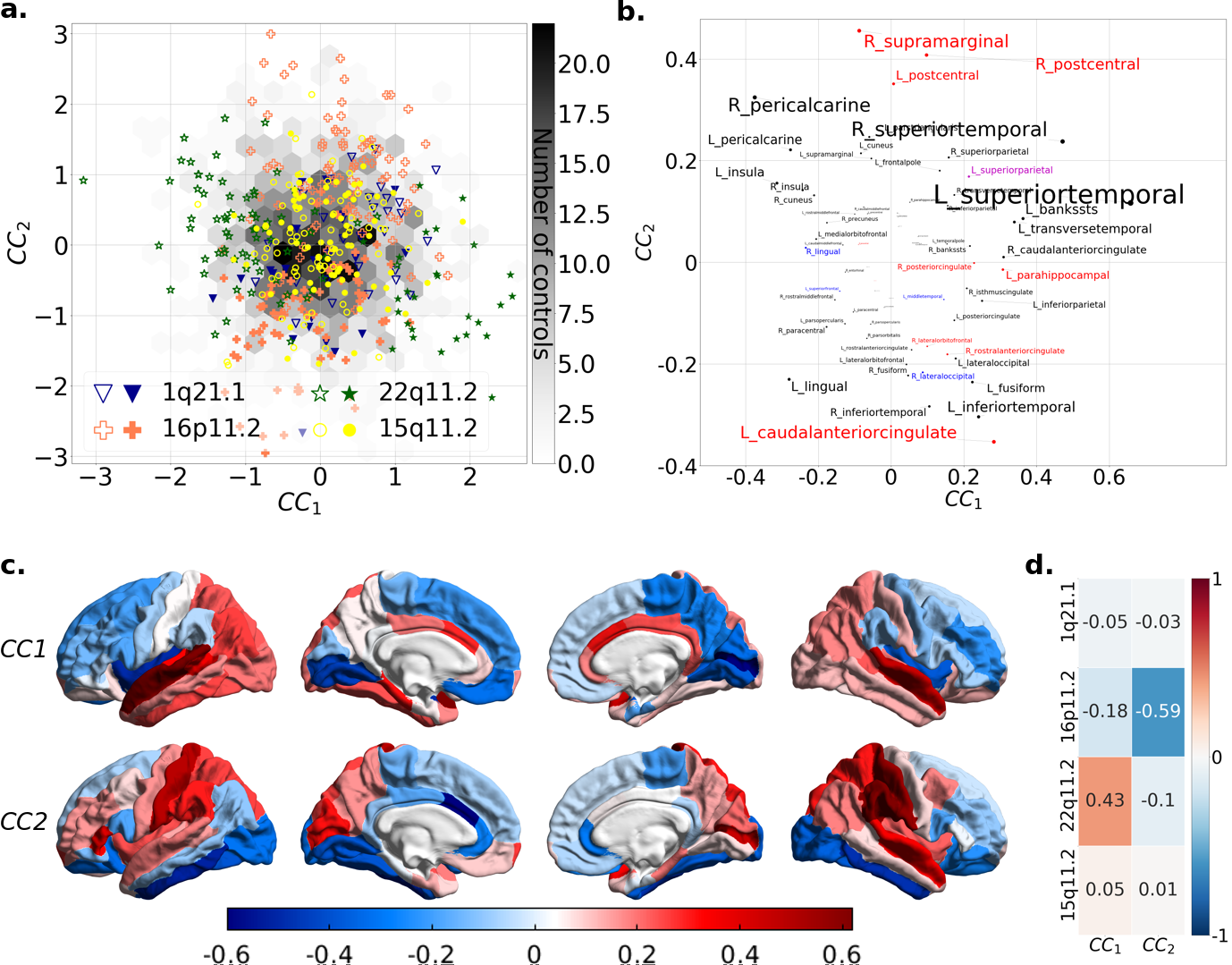


## Supplementary Figure 15: CCA analysis across 4 Genomic Loci, Cortical Thickness

Legend: (a) Scatterplot showing the position of each of the 468 carriers of 8 different CNVs along 2 Canonical Correlation (CC) dimensions established using 68 Freesurfer regions of CNV carriers. Cortical Thickness (CT) was calculated using Freesurfer recon-all and was adjusted for mean CT, age, age^2^, sex, and site. The empty and full symbols represent deletions and duplication respectively. The grey hexagonal bin plot represents the frequency of controls (n=1252). Controls were not used to compute the CCA and were projected post hoc on the 2 dimensions using CCA prediction. X and Y axis values: z-scores of regional volumes. (b) Loading of Freesurfer Regions of Interests (ROIs) on the 2 CC dimensions. The font size is correlated to the region's contribution to CC dimensions. ROI names are color-coded as being part of the deletion (red), duplication (blue) and both deletion and duplication (magenta) convergence patterns. (c) CCA dimensions 1 and 2 projected on the brain. Brain regions most strongly associated with CCA dimensions 1 and 2. The darker the red or blue color, the stronger the positive or negative association with the CCA dimensions. (d) Loading of the first and second CCA dimensions on 4 CNV genomic loci. Values are CCA loading magnitudes and represent the contribution of a CNV locus to the canonical dimension. CCA was calculated using 449 CNV carriers without controls and identified 2 dimensions (r=0.69, 0.65 statistically significant at p-value<0.05). Top 20 ROI loadings (absolute values) for CC1 and CC2 are listed in eTable 7.


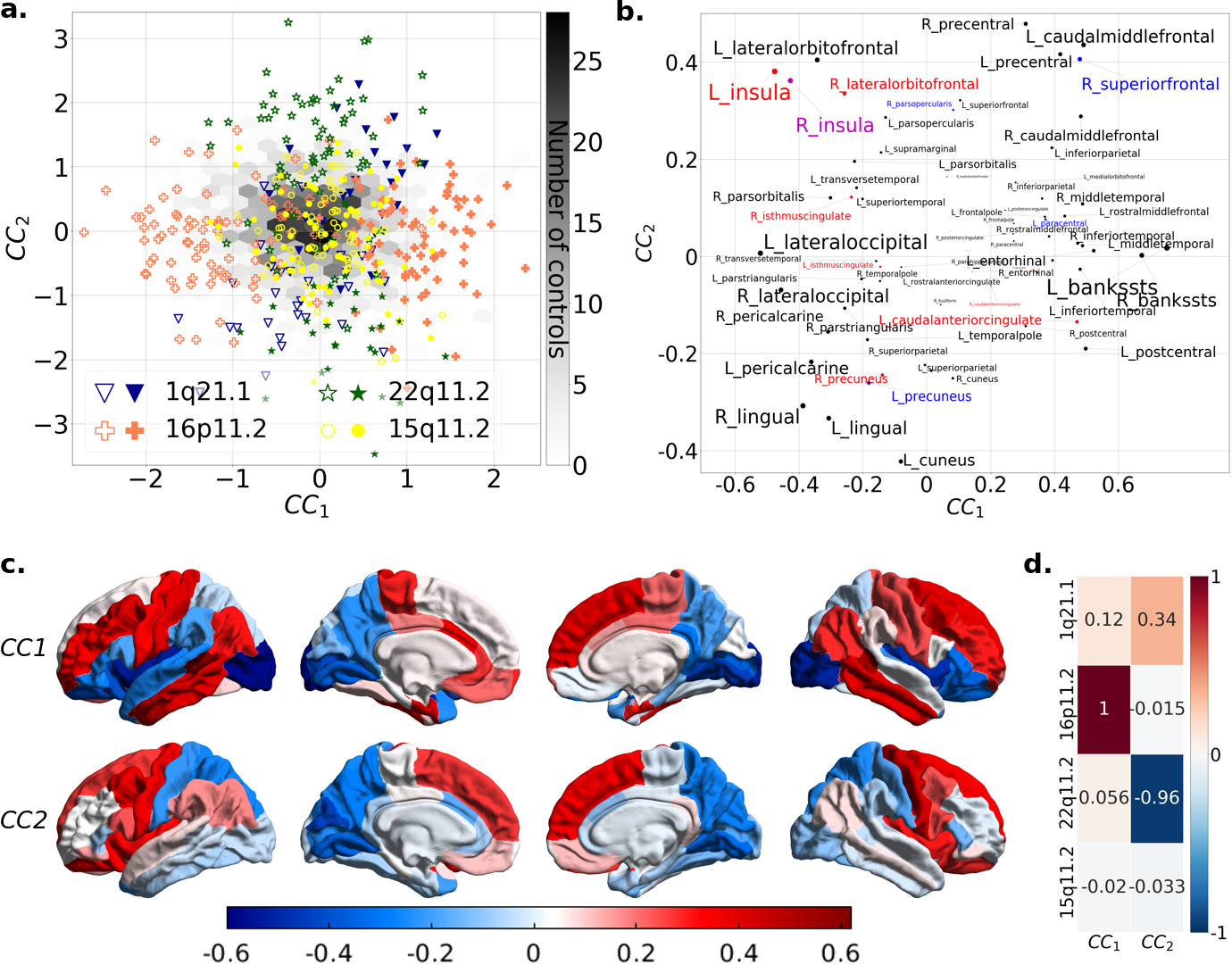


## Supplementary Figure 16: CCA analysis across 4 Genomic Loci, Surface Area

Legend: (a) Scatterplot showing the position of each of the 468 carriers of 8 different CNVs along 2 Canonical Correlation (CC) dimensions established using 68 Freesurfer regions of CNV carriers. Surface Area (SA) was calculated using Freesurfer recon-all and were adjusted for mean total SA, age, age^2^, sex and site. The empty and full symbols represent deletions and duplication respectively. The grey hexagonal bin plot represents the frequency of controls (n=1252). Controls were not used to compute the CCA and were projected post hoc on the 2 dimensions using CCA prediction. X and Y axis values: z-scores of regional volumes. (b) Loading of Freesurfer Regions of Interests (ROIs) on the 2 CC dimensions. The font size is correlated to the region's contribution to CC dimensions. ROI names are color coded as being part of the deletion (red), duplication (blue) and both deletion and duplication (magenta) convergence patterns. (c) CCA dimensions 1 and 2 projected on the brain. Brain regions most strongly associated with CCA dimensions 1 and 2. The darker the red or blue color, the stronger the positive or negative association with the CCA dimensions. (d) Loading of the first and second CCA dimensions on 4 CNV genomic loci. Values are CCA loading magnitudes and represent the contribution of a CNV loci to the canonical dimension. CCA was calculated using 449 CNV carriers without controls and identified 2 dimensions (r=0.79, 0.67 statistically significant at p-value<0.05). Top 20 ROI loadings (absolute values) for CC1 and CC2 are listed in eTable 8.

## References

[1. Lin, A. *et al.* Mapping 22q11.2 Gene Dosage Effects on Brain Morphometry. *J. Neurosci.* **37**, 6183–6199 (2017).](https://www.zotero.org/google-docs/?8YA00S)

[2. Maillard, A. M. *et al.* The 16p11.2 locus modulates brain structures common to autism, schizophrenia and obesity. *Mol. Psychiatry* **20**, 140–147 (2015).](https://www.zotero.org/google-docs/?8YA00S)

[3. Qureshi, A. Y. *et al.* Opposing Brain Differences in 16p11.2 Deletion and Duplication Carriers. *J. Neurosci.* **34**, 11199–11211 (2014).](https://www.zotero.org/google-docs/?8YA00S)

[4. Martin-Brevet, S. *et al.* Quantifying the Effects of 16p11.2 Copy Number Variants on Brain Structure: A Multisite Genetic-First Study. *Biol. Psychiatry* **84**, 253–264 (2018).](https://www.zotero.org/google-docs/?8YA00S)

[5. Miller, K. L. *et al.* Multimodal population brain imaging in the UK Biobank prospective epidemiological study. *Nat. Neurosci.* **19**, 1523–1536 (2016).](https://www.zotero.org/google-docs/?8YA00S)

[6. Sudlow, C. *et al.* UK Biobank: An Open Access Resource for Identifying the Causes of a Wide Range of Complex Diseases of Middle and Old Age. *PLOS Med.* **12**, e1001779 (2015).](https://www.zotero.org/google-docs/?8YA00S)

[7. Huguet, G. *et al.* Measuring and Estimating the Effect Sizes of Copy Number Variants on General Intelligence in Community-Based Samples. *JAMA Psychiatry* **75**, 447–457 (2018).](https://www.zotero.org/google-docs/?8YA00S)

[8. Douard, E. *et al.* Effect Sizes of Deletions and Duplications on Autism Risk Across the Genome. *Am. J. Psychiatry* **178**, 87–98 (2020).](https://www.zotero.org/google-docs/?8YA00S)

[9. Ashburner, J. & Friston, K. J. Unified segmentation. *NeuroImage* **26**, 839–851 (2005).](https://www.zotero.org/google-docs/?8YA00S)

[10. Lorio, S. *et al.* New tissue priors for improved automated classification of subcortical brain structures on MRI. *Neuroimage* **130**, 157–166 (2016).](https://www.zotero.org/google-docs/?8YA00S)

[11. Ashburner, J. A fast diffeomorphic image registration algorithm. *NeuroImage* **38**, 95–113 (2007).](https://www.zotero.org/google-docs/?8YA00S)

[12. Desikan, R. S. *et al.* An automated labeling system for subdividing the human cerebral cortex on MRI scans into gyral based regions of interest. *NeuroImage* **31**, 968–980 (2006).](https://www.zotero.org/google-docs/?8YA00S)

[13. Worsley, K. J. *et al.* SurfStat: A Matlab toolbox for the statistical analysis of univariate and multivariate surface and volumetric data using linear mixed effects models and random field theory. *Neuroimage* (2009) doi:10.1016/S1053-8119(09)70882-1.](https://www.zotero.org/google-docs/?8YA00S)

[14. Cohen, J. *Statistical power analysis for the behavioral sciences*. (Psychology Press, 1988).](https://www.zotero.org/google-docs/?8YA00S)

[15. Alexander-Bloch, A. *et al.* On testing for spatial correspondence between maps of human brain structure and function. *NeuroImage* **178**, 540–551 (2018).](https://www.zotero.org/google-docs/?8YA00S)

[16. Reardon, P. K. *et al.* Normative brain size variation and brain shape diversity in humans. *Science* **360**, 1222–1227 (2018).](https://www.zotero.org/google-docs/?8YA00S)

[17. Smith, S. M. *et al.* A positive-negative mode of population covariation links brain connectivity, demographics and behavior. *Nat. Neurosci.* **18**, 1565–1567 (2015).](https://www.zotero.org/google-docs/?8YA00S)

[18. Wang, H.-T. *et al.* Finding the needle in high-dimensional haystack: A tutorial on canonical correlation analysis. *NeuroImage* (2020).](https://www.zotero.org/google-docs/?8YA00S)

[19. Kernbach, J. M. *et al.* Subspecialization within default mode nodes characterized in 10,000 UK Biobank participants. *Proc. Natl. Acad. Sci.* **115**, 12295–12300 (2018).](https://www.zotero.org/google-docs/?8YA00S)

[20. Bzdok, D. & Yeo, B. T. T. Inference in the age of big data: Future perspectives on neuroscience. *NeuroImage* **155**, 549–564 (2017).](https://www.zotero.org/google-docs/?8YA00S)
